# Functional study of *Bergeyella cardium* KP-43 subfamily peptidases as putative T9SS cargo

**DOI:** 10.1038/s42003-025-07996-y

**Published:** 2025-04-09

**Authors:** Tian Li, Yiwen Gao, Xiaoyue Zhang, Yuxiao Zhao, Fuyao Hu, Wei Li, Lixiang Li, Hongwei Pan, Yi Zhang, Ying Chen

**Affiliations:** 1 Department of Clinical Laboratory, Qilu Hospital of Shandong University, Jinan, 250012 Shandong China; 2 Department of Gastroenterology, Qilu Hospital of Shandong University, Jinan, 250012 Shandong China; 3 Shandong Engineering Research Center of Biomarker and Artificial Intelligence Application, Jinan, 250012 Shandong China; 4 Clinical Molecular Diagnostics Institute of Shandong University, Jinan, 250012 Shandong China

**Keywords:** Bacterial pathogenesis, Bacteriology

## Abstract

*Bergeyella cardium* causes infections in human organs. However, the mechanism of the virulence of *B. cardium* is unclear. Peptidases are important virulence factors in bacterial pathogens. Here, we identified three KP-43 subfamily peptidases, SpBcA, SpBcB and SpBcC, which are putative T9SS cargo proteins, and analyzed their protease activity. SpBcA and SpBcB are active in vitro and contain a propeptide that passes through the active site of the S8 peptidase domain and inhibits its activity. SpBcA activates itself by cleaving the propeptide at N102 within the TSNA (100–103) peptide and a putative cleavage site at 116–120 (TSPGL). Additionally, SpBcA degrades host defense molecules, fibrinogen, antimicrobial peptide LL-37 and gelatin in vitro and induces cell death in vivo, suggesting its role as a virulence factor. This study revealed the self-cleavage regulatory mechanism of SpBcA and provided a basis for studying how *B. cardium* uses peptidases as virulence factors in vivo.

## Abstract (web summary)

A study reveals the regulatory mechanism of *Bergeyella cardium* peptidase SpBcA by self-cleavage and the role of SpBcA in degrading host defense proteins and inducing cell death, elucidating peptidase-driven virulence strategies of *B. cardium*.

## Introduction

*Bergeyella* sp. is a genus of nonfermenting gram-negative aerobic bacteria belonging to the Flavobacteriaceae family. It can cause infectious diseases, such as bacteremia, pneumonia, meningitis, cellulitis, abscesses, and infective endocarditis^1–6^. Additionally, its colonization is potentially associated with IgA nephropathy and cancer^7–11^. However, owing to difficulties in culturing and isolating bacteria in this genus, our current understanding of this genus is limited primarily to case reports, and little is known about the virulence factors in *Bergeyella* sp.

The recently discovered type IX secretion system (T9SS), which is found only in species of the phylum Bacteroidota, is associated with the secretion of proteins that are virulence factors for many pathogens^12–15^. Some of the best-studied proteins secreted by the T9SS are peptidases. Peptidases are recognized as important virulence factors in different pathogens and can degrade protein components of infected tissues, facilitate host colonization and dissemination of bacteria in the host, or protect pathogens from immune system responses^16–18^. In *Riemerella anatipestifer*, an important T9SS effector, SspA, was identified as a secreted subtilisin-like serine protease. SspA is associated with virulence and host complement evasion. Thus, this protease is thought to play an important role in *R. anatipestifer* virulence^19^.

Proteases are typically produced and secreted as inactive precursors and require cleavage for activation. Subtilisin is one of the best studied proteases. Previous studies have shown that most subtilisins are produced in their zymogen form, likely to prevent premature protease activity that could lead to improper protein activation, sorting, or degradation^20,21^. The inhibitor I9 domain is found in the N-terminus of most subtilisin proteases as the inhibitory propeptide domain. This sequence traverses the active site of the subtilisin protease, serving as an intramolecular chaperone and a transient inhibitor of protease activity. In certain biological processes, subtilisin can autologously release its propeptide domain under specific conditions, thereby becoming a mature enzyme^21–24^.

Previously, Pan et al. isolated and identified a *Bergeyella cardium* strain named *B. cardium* HPQL from a positive blood culture of a patient with infective endocarditis^25^ and obtained its whole-genome sequence^25^. In this study, we aimed to identify the virulence factors potentially secreted by the T9SS system in *B. cardium* to provide insight into the mechanisms involved in the pathogenesis of *Bergeyella* sp. As the serine protease domain SspA, an important T9SS effector, plays an important role in the virulence of *R. anatipestifer*^19^, which is phylogenetically close to *B. cardium*^26^, we looked for proteins encoding the serine protease domain and the C-terminal structural domain (CTD), the featured domain for the T9SS cargo, as potential virulence factors.

On the basis of whole-genome analysis, we identified and chose three proteins encoding putative serine protease domains and CTDs, namely, QHN65200.1, QHN65156.1 and QHN66091.1, here termed SpBcA, SpBcB and SpBcC, respectively, for serine peptidases from *B. cardium*. All three proteins contain a subtilisin-like serine protease domain, belonging to the KP-43 subfamily of the S8 family of peptidases, at the N-terminus and a CTD at the C-terminus, indicating that they are likely peptidases secreted by the T9SS. Structure prediction and comparison indicate that the structures of the three proteins differ from each other and from those of other reported peptidases. We expressed and purified these peptidases and analyzed their protease activity and virulence. On the basis of the structural and biochemical analyses, we propose a self-cleavage regulatory mechanism for the protease activity of SpBcA.

## Results

### Identification of three peptidases as potential cargo for the T9SS

To study how *B. cardium* exerts its virulence, we searched for proteins in the National Center for Biotechnology Information (https://www.ncbi.nlm.nih.gov/) using “*Bergeyella cardium* HPQL and S8 family of peptidase” as input and identified six proteins, which are listed in Supplementary Table 1 (Supplementary Table 1). The sequences of QHN65150.1 and QHN65200.1 are identical; QHN65828.1 and QHN64674.1 contain only the S8 peptidase domain. Therefore, we chose three proteins, QHN65200.1, QHN65156.1 and QHN66091.1, which contain both the S8 peptidase domain and the CTD domain, and named them SpBcA, SpBcB and SpBcC, respectively.

**Fig. 1:**
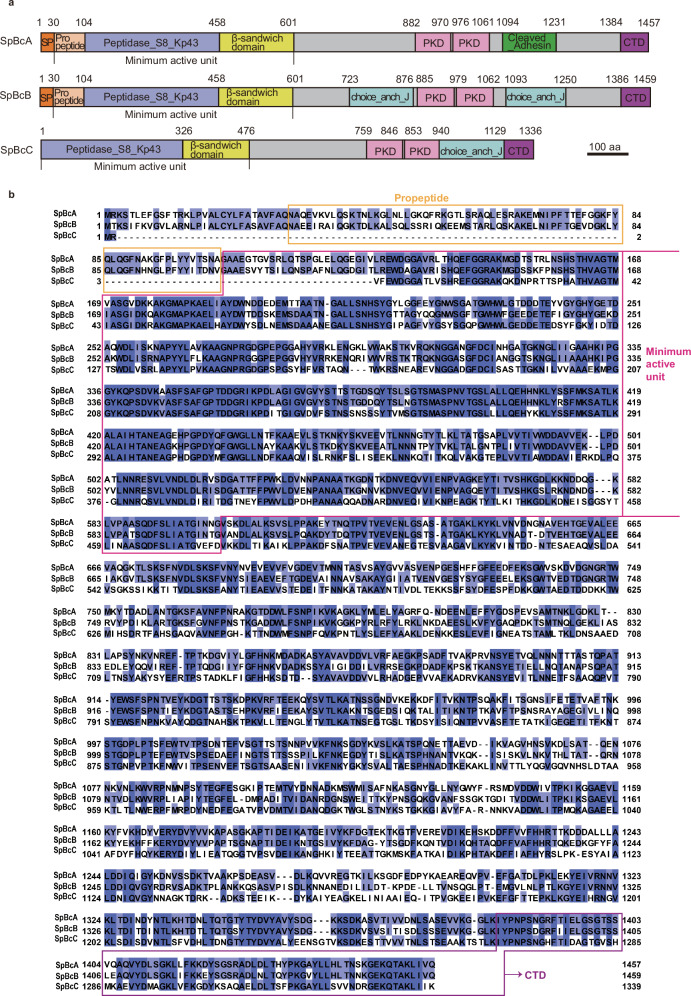
Domain organization and sequence alignment of SpBcA, SpBcB and SpBcC. **a** Domain organization of SpBcA, SpBcB and SpBcC. The color scheme is as follows: orange, signal peptidase (SP); light orange, propeptide; light blue, peptidase S8 family domain in kp43 proteases (peptidase_S8 domain); yellow, β-sandwich domain; pink, polycystic kidney disease 1 (PKD1) domain; green, cleaved adhesin domain; magenta, the C-terminal sorting domain (CTD) of the T9SS; cyan, choice-of-anchor j domain (choice-of-anchor J). **b** Multiple sequence alignment of SpBcA, SpBcB and SpBcC by Jalview. Residues with identities above 60% are colored in blue. A deeper color represents a higher percentage of identity.

The three peptidases seem to be paralogs and share some common domains, including the peptidase_S8 domain, the PKD (polycystic kidney disease 1) domain and the CTD domain (Fig. 1a). Peptidases_S8_Kp43_proteases are members of the peptidase S8 or subtilase clan of proteases, which share an Asp/His/Ser catalytic triad^27^. The functional CTD domain targets proteins to T9SS secretion systems and is cleaved by a C-terminal signal peptidase after secretion^28^. Whether the CTDs of SpBcA, B and C are functional remains to be investigated. The PKD domain is associated with polycystic kidney disease 1 and plays a role in cell‒cell/matrix interactions^29,30^. In addition to the shared domains, the peptidases contain specific additional domains. SpBcA contains a cleaved adhesin domain, which is a family of bacterial protein modules thought to function in cell adhesion, cell lysis and carbohydrate binding^31,32^. SpBcB and SpBcC contain a Choice_anch_J superfamily domain (Fig. 1a).

Additionally, both SpBcA and SpBcB contain an SEC-dependent signal peptide at the N-terminus that allows them to translocate across the inner membrane via the SEC mechanism, which is characteristic of effector proteins^33^, followed by an N-terminal domain with unknown function. However, this N-terminal sequence is not predicted in the SpBcC sequence (Fig. 1a).

Sequence alignment revealed that the three peptidases are similar to each other, especially in the peptidase domain, whereas the similarity is lower in the variable domains (Fig. 1b). Structure prediction further revealed that the N-termini of SpBcA, SpBcB and SpBcC share similar folds. On the basis of this information, we believe that SpBcA, B and C are paralogs with potentially different biological functions and are potential cargos for the T9SS. Furthermore, we compared *B. cardium* peptidases with S8 peptidases from other species, including *R. anatipestifer*, *Porphyromonas gingivalis*, and *Bacillus* sp. (Supplementary Fig. 1). The results revealed that the structural domains of the S8 peptidases are relatively conserved and that the *B. cardium* peptidases are more related to peptidases from *R. anatipestifer* bacteria.

### Expression and purification of peptidases guided by structure prediction

**Fig. 2:**
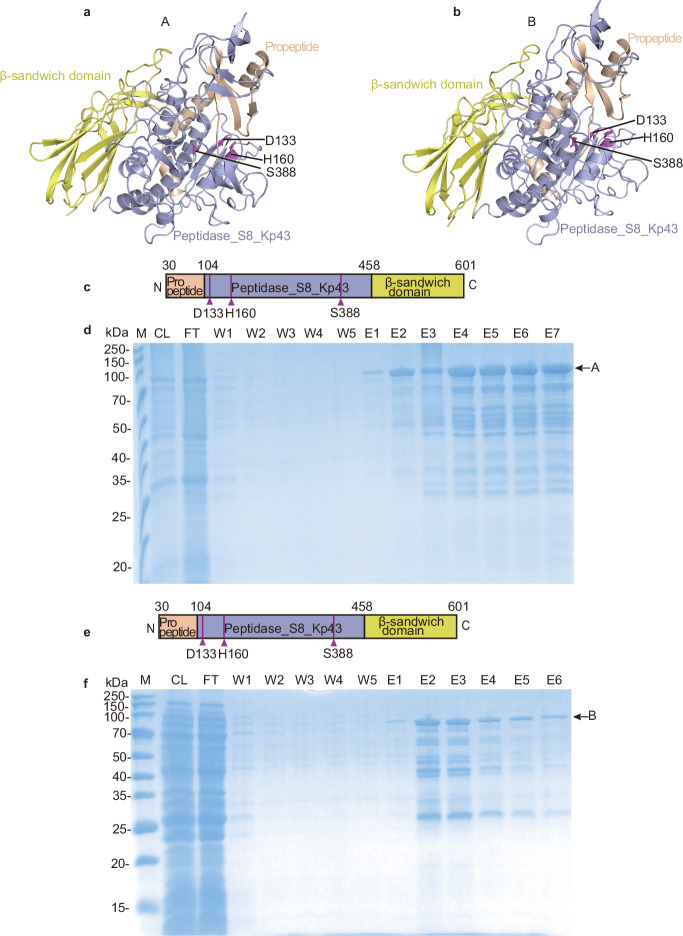
Expression and purification of the functional domains of the SpBcA and SpBcB peptidases guided by structure prediction. **a**, **b** The predicted structures of SpBcA (30–601 aa) (A) and SpBcB (30–601 aa) (B), containing the propeptide domain, S8 peptidase domain and auxiliary β-sandwich domain, are shown as cartoon representations and are colored light orange, light blue and yellow, respectively. Residues at the active center are shown as stick representations and colored in purple. **c**, **e** Domain organization of A and B. **d**,**f** Purification of peptidases A and B. CL cell lysate, FT flow through, W1–W5 wash fractions, E1–E7 elution fractions.

To further characterize these peptidases, we cloned SpBcA, B and C and expressed them in *Escherichia coli*. We first tried to express and purify full-length proteins; however, only SpBcC could be expressed and purified in very low amounts and was inactive in vitro (Supplementary Fig. 2a, b). SpBcA and SpBcB were not soluble. To obtain soluble proteins for biochemical studies, we designed truncation variants of the proteins on the basis of structure prediction by trRosseta (Supplementary Fig. 2c–e), consisting of the predicated S8 peptidase domain and its auxiliary domain^34^, namely, SpBcA (30–601 aa), SpBcB (30–601 aa) (Fig. 2a–c, e) and SpBcC (1–476 aa), and tried to express and purify them in vitro. SpBcA (30–601 aa) and SpBcB (30–601 aa) were expressed and purified successfully (Fig. 2d, f), whereas SpBcC (1–476 aa) could not be expressed. In the following text, unless otherwise specified, we refer to SpBcA (30–601 aa) and SpBcB (30–601 aa) as A and B, respectively, for simplicity.

During purification, we detected additional protein bands in the elution fractions of the GST batch purification of A and B. Mass spectrometry analysis of these bands revealed that they were degradation products of A (Supplementary Fig. 3a, b) and B (Supplementary Fig. 4a–c), respectively. The peptides from the B sample were further searched against the protein database of *E.coli* and revealed almost no matching proteins (Supplementary Fig. 5a–d). This ruled out the possibility of protein contamination from *E. coli* and implied that A and B might be active.

### Characterization of peptidase A activity in vitro

**Fig. 3:**
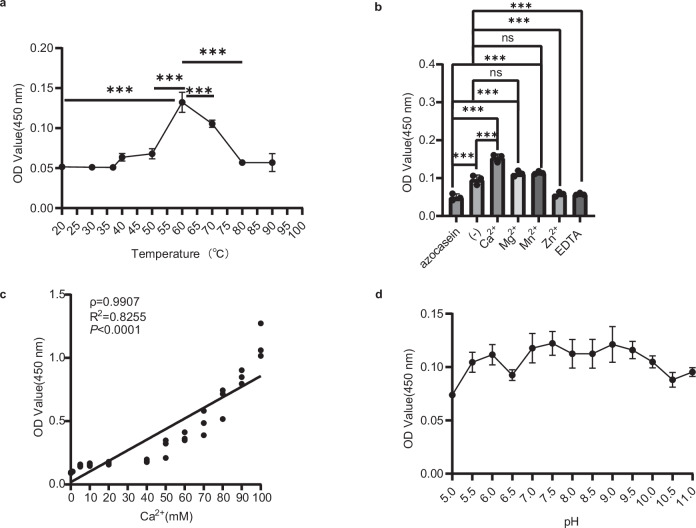
Optimal reaction conditions for SpBcA (30–601 aa) using azocasein as the substrate. **a** Analysis of the optimal temperature for protease activity. **b** Analysis of the effect of cations at 60 °C. **c** Analyze the correlation between Ca^2+^ concentration and protein activity. **d** Analysis of the optimal pH at 60 °C. The vertical axis indicates the UV absorbance of the proteolysis product at 450 nm. The error bars indicate the standard deviations calculated from three experiments (*n* = 3). The data are shown as the means ± SD. Statistical significance is indicated by *p* values, which were assessed using one-way ANOVA: ns, not significant; *, *p* < 0.05; **, *p* < 0.01; ***, *p* < 0.001. In panel (**c**), the relationship between Ca^2+^ and the proteolysis products was assessed by Spearman correlation coefficient.

To test whether peptidase A was truly active, we performed protease assays under different reaction conditions using azocasein, a chromogenic derivative of casein, as the substrate. The degradation of azocasein by proteases yields TCA-soluble azopeptides with high UV absorbance at 450 nm, which are used as indicators of protease activity. We first evaluated the optimal temperature for peptidase A by performing a protease assay at a temperature range of 20–90 °C. The assay revealed that the optimal temperature for peptidase A activity is 60 °C (Fig. 3a). We further tested the effect of cations by adding Ca²⁺, Mg²⁺, Mn²⁺, Zn²⁺ and EDTA (Fig. 3b). The results indicated that the protease activity of A was stimulated by Ca²⁺. Mg²⁺ and Mn²⁺ did not stimulate the activity, whereas Zn²⁺ and EDTA inhibited the activity of A. The activity of the protein increased with increasing calcium ion concentration (Fig. 3c). Similarly, we tested the optimal pH for A by performing protease assays over a pH range of 5 to 11. The optimal pH for A seems to be 7.5 but requires further investigation, as the activity of A remains quite similar between pH 7 and pH 9.5 (Fig. 3d).

### Verification of the Peptidase A and B active centers

**Fig. 4:**
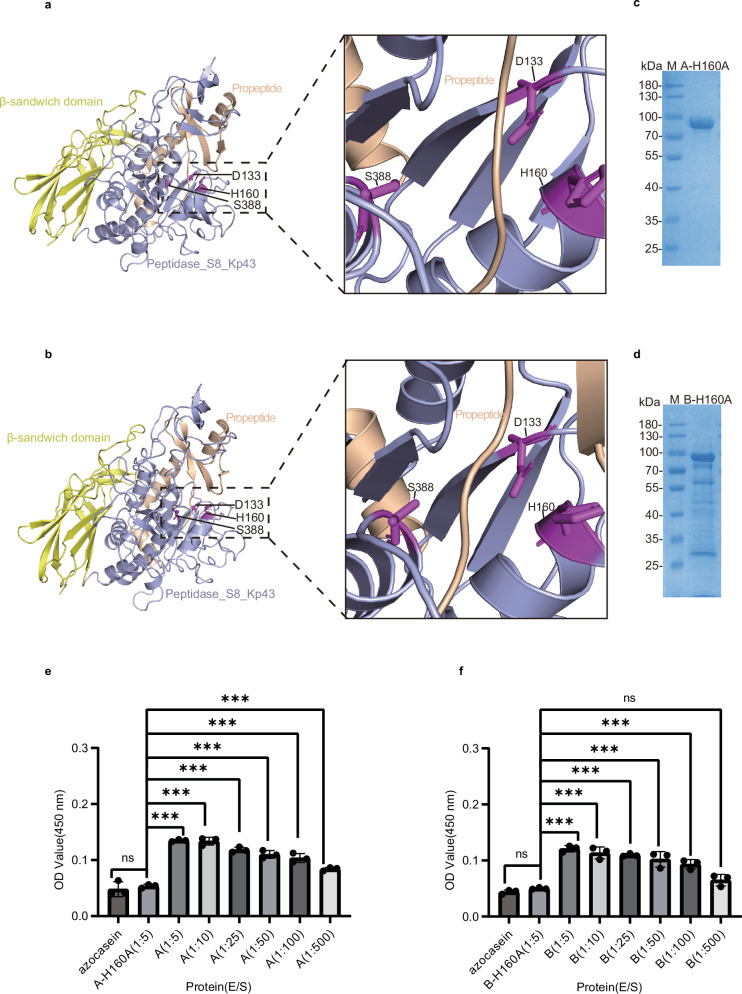
Activity of the proteases SpBcA (30–601 aa) and SpBcB (30–601 aa), along with the construction of their mutants and determination of activity. Predicted structures of SpBcA (30–601 aa) (A) (**a**) and SpBcB (30–601 aa) (B) (**b**). The region around the active sites is shown in a zoomed-in view. The catalytic triad residues D133, H160 and S388 are shown as stick representations and are colored purple. The color scheme of the structure is the same as that in Fig. 2. SDS‒PAGE analyses of a purified A catalytic mutant, A-H160A (**c**) and a B catalytic mutant, B-H160A (**d**). Protease assays for A (**e**) and B (**f**) using azocasein as the substrate. The vertical axis indicates the UV absorbance of the proteolysis product at 450 nm. The horizontal axis shows the names of the protein samples involved and the enzyme-to-substrate final concentration ratio (E/S). The error bars indicate the standard deviations calculated from three experiments (*n* = 3). The data are shown as the means ± SD. Statistical significance is indicated by *p* values, which were assessed using one-way ANOVA: ns not significant, * (*p* < 0.05), ** (*p* < 0.01), *** (*p* < 0.001).

According to the protein sequences, peptidases A and B contain a conserved catalytic triad structure (Asp133, His160, and Ser388), similar to subtilisin and other members of the S8 protease family (Fig. 4a, b). To verify the active center, we generated putative catalytic mutants of A and B (A-H160A and B-H160A, Fig. 4c, d) and performed protease assays with wild-type A and B and the putative catalytic mutants (H160A) at 60 °C with 5 mM CaCl_2_ in the reaction using azocasein as a substrate (Fig. 4e, f). The results revealed that wild-type A and B degraded azocasein and that the amount of product produced was positively related to the concentration of the protease, whereas the catalytic mutants failed to degrade azocasein, indicating the loss of peptidase activity. These results verified the active center in peptidases A and B. Additionally, A seems to have higher activity than B does, since at an enzyme-to-substrate ratio of 1:500, the activity of A, but not B, could be detected (Fig. 4e, f). Protease assays using fibrinogen and casein as substrates also revealed that wild-type A was active and that A-H160A was inactive, which is consistent with the observations with azocasein (Supplementary Fig. 6a, b).

### Self-cleavage activates peptidases A and B

**Fig. 5:**
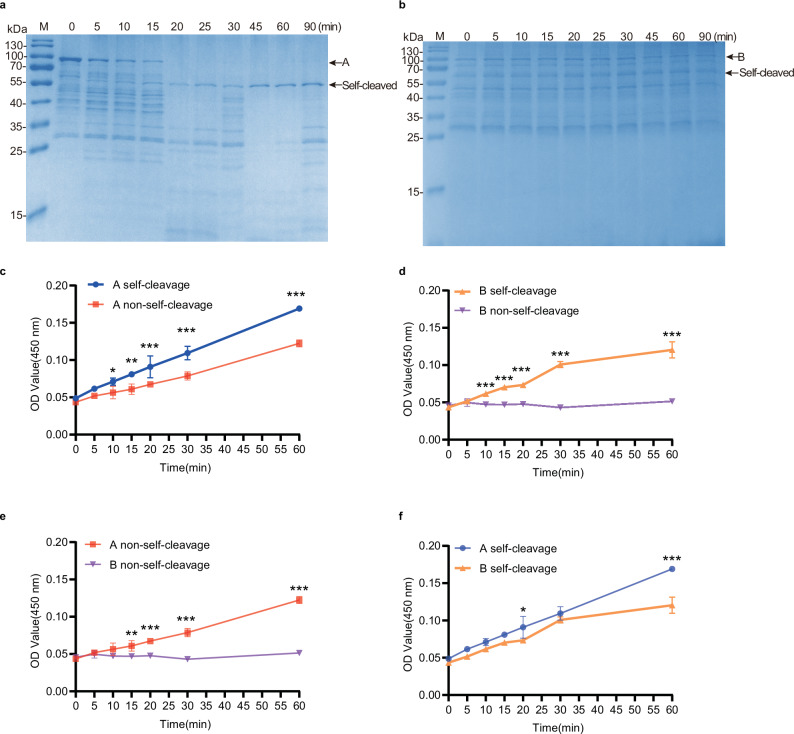
Self-cleavage activates the activity of the two peptidases SpBcA (30–601 aa) and SpBcB (30–601 aa). Self-cleavage of A and B over time. Peptidases A (**a**) and B (**b**) (0.087 µg/µL final concentration) were preincubated in 115 μL buffer (100 mM Tris-HCl, 150 mM NaCl, 5 mM CaCl_2_ and ddH_2_O, pH 7.4) at 37 °C. Comparison of the activities of peptidases A (**c**) and B (**d**) with and without self-cleavage over time when azocasein was used as the substrate at 60 °C. The data are shown as the means ± SD. The points are connected with lines to show the trend over time. The error bars indicate the standard deviations calculated from three experiments (*n* = 3). Statistical significance is indicated by *p* values, which were assessed using two-way ANOVA: * (*p* < 0.05), ** (*p* < 0.01), *** (*p* < 0.001). Replot the data shown in (**c**) and (**d**) to compare the activities of A and B in the absence (**e**) and presence of self-cleavage (**f**).

We also performed protease assays using casein as the substrate at 37 °C. During these assays, we noticed downshifting of the band corresponding to peptidase A via SDS‒PAGE, indicating that proteolysis decreased the size of peptidase A, whereas no clear shift was observed for peptidase B (Supplementary Fig. 6c, d). Since the S8 serine peptidase subtilisin exists as a pro-protease with an N-terminal leading sequence that inhibits its protease activity^21–24^, and subtilisin is activated upon self-cleavage, we suspected that peptidase A might also undergo self-cleavage. To test this hypothesis, we performed a self-cleavage assay using GST-tagged A as the substrate and collected samples at various time points. Indeed, we observed a clear shift in the protein bands for A after incubation (Fig. 5a, Supplementary Fig. 3c). Mass spectrometry of the downshifted band confirmed that this band was indeed A (Supplementary Fig. 3c, d). The self-cleavage of A was completed after approximately 20 min (Fig. 5a). We performed similar self-cleavage experiments with peptidase B and observed no clear specific band shift (Fig. 5b). Although no significant self-cleavage bands were observed for peptidase B, we hypothesized that it might possess potential self-cleavage capability based on its structural homology with peptidase A. Therefore, we also conducted in vitro biochemical assays to investigate this possibility.

Next, we tested whether A and B are activated by self-cleavage. We performed protease assays using azocasein as a substrate at 60 °C and compared the activity of peptidases A and B, with or without preincubation, before adding azocasein (self-cleavage) (Fig. 5c–d). We performed two reactions in parallel: in reaction 1, peptidases A and B directly reacted with azocasein as a control; in reaction 2, peptidases A and B were preincubated in the reaction system for 20 min to complete self-cleavage before azocasein was added. The results showed that after self-cleavage, the activity of A was significantly greater than that without self-cleavage (Fig. 5c). The activity of B was also significantly (*p* < 0.001) increased after self-cleavage (Fig. 5d). This implies that self-cleavage indeed activated peptidase A and might also activate peptidase B.

We further compared the activities of peptidases A and B by plotting their data on the same graph as the same data shown in Fig. 5c, d. The graphs revealed that without self-cleavage, A was much more active than B. However, activation by self-cleavage reduced the activity difference between A and B (Fig. 5e, f). Protease assays using casein as a substrate also revealed that self-cleavage can promote the activity of A, but its effect on B is not very obvious due to insufficient sensitivity (Supplementary Fig. 7a–d). Since self-cleavage enhanced the activity of the peptidases, in subsequent experiments, we preincubated the peptidases in the absence of substrates to achieve full activation before the protease assay.

### Reciprocal cleavage between peptidases A and B

**Fig. 6:**
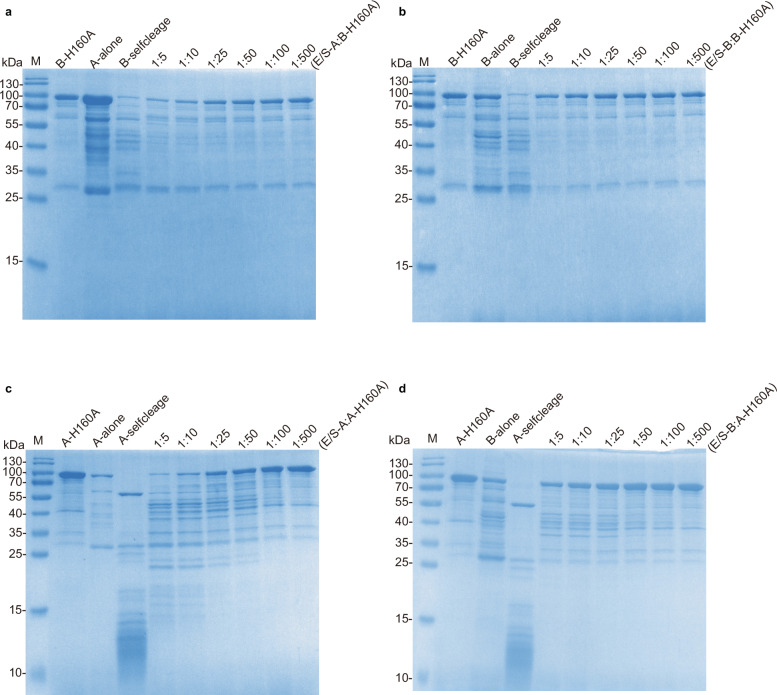
Reciprocal cleavage between the peptidases SpBcA (30–601 aa) and SpBcB (30–601 aa). Cleavage of the B catalytic mutant (B-H160A, final concentration of 0.26 µg/µL) by wild-type peptidase A (**a**) or B (**b**). The assay was performed in buffer containing 100 mM Tris-HCl, 150 mM NaCl, 5 mM CaCl_2_ and ddH_2_O, pH 7.4, with a final total volume of 115 μL, with different enzyme-to-substrate final concentration ratios (E/S): 1:5, 1:10, 1:25, 1:50, 1:100, and 1:500. B-H160A, A-alone, B-alone and B-self-cleavage were loaded onto the gel as controls. B-self-cleavage means that the B protein alone was incubated in the assay buffer at 37 °C for 20 min. Cleavage of A catalytic mutant (A-H160A, final concentration of 0.26 µg/µL) by wild-type peptidase A (**c**) or B (**d**). The rest of the steps are the same as those described above for points (**a**) and (**b**).

As both A and B are proteases that may coexist in the cell, we next investigated whether they can cleave each other. Since our aim was to explore the potential activity of the protein in vivo, we performed protease assays at 37 °C instead of 60 °C to mimic the optimal physiological temperature of human cells. Additionally, the peptidases were preincubated for 20 min prior to the addition of substrates for self-cleavage. We first performed reciprocal cleavage of wild-type A and B (Supplementary Fig. 7e, f), but the results were difficult to interpret because A and B degrade each other and themselves. Therefore, we used A and B to cleave the catalytic mutants B-H160A and A-H160A, respectively. The results showed that both A and B could use A-H160A or B-H160A as substrates, and A exhibited higher activity than B did (Fig. 6a–d).

Curiously, when the catalytic mutant of A (A-H160A) was used as the substrate, A could not cleave the mutant at the specific self-cleavage site, as indicated by the absence of a specific band shift (Fig. 6c). It is possible that the active site residues are important not only for cleavage activity but also for defining the primary cleavage site. Since peptidase B exhibited no specific band after self-cleavage, we next focused on peptidase A in our subsequent study on the regulatory mechanism of peptidase activation by self-cleavage.

### Regulatory mechanism of peptidase activity and identification of the cleavage site of A

**Fig. 7:**
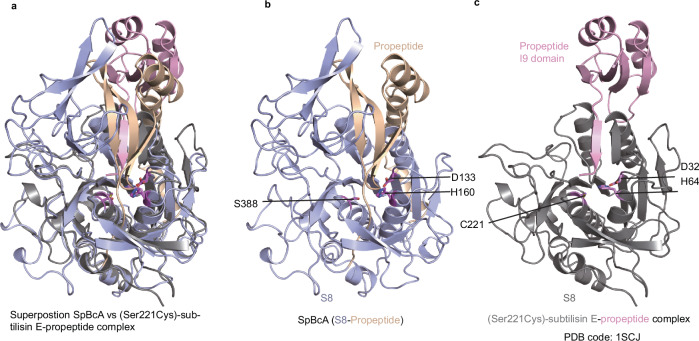
Comparison of the SpBcA N-terminal propeptide and the subtilisin I9 domain. **a** Superposition of the structures of SpBcA and the (Ser221Cys)-subtilisin E-propeptide complex (PDB code: 1SCJ). The structure of the SpBcA S8-peptidase domain was superposed with the (Ser221Cys)-subtilisin E-propeptide complex (PDB code: 1SCJ). The structure of SpBcA is colored in the same scheme as in Fig. 2. The I9 domain and the S8 domain of 1SCJ are colored in pink and gray, respectively. The two structures are shown side by side in the superposed orientation in panels (**b**) and (**c**). **b** The structure of the SpBcA N-terminal propeptide and the S8 peptidase domain. **c** The structure of the (Ser221Cys)-subtilisin E-propeptide complex.

To study the mechanism of activation by self-cleavage, we further analyzed the sequence and structure of A. A contains an N-terminal sequence preceding the peptidase domain, a propeptide that corresponds to the N-terminal propeptide of subtilisin, the I9 domain. However, superposition of the structures of A and the (Ser221Cys)-subtilisin E-propeptide complex shows that the A propeptide has no sequence or structural similarity to the I9 domain^35^ (Fig. 7a–c). According to the structure prediction, the N-terminal domain of peptidase A contains a peptide that passes through the active site of the peptidase. Thus, we hypothesize that the N-terminal sequence likely inhibits the activity of peptidase A and that cleaving this domain should eliminate this inhibition.

**Fig. 8:**
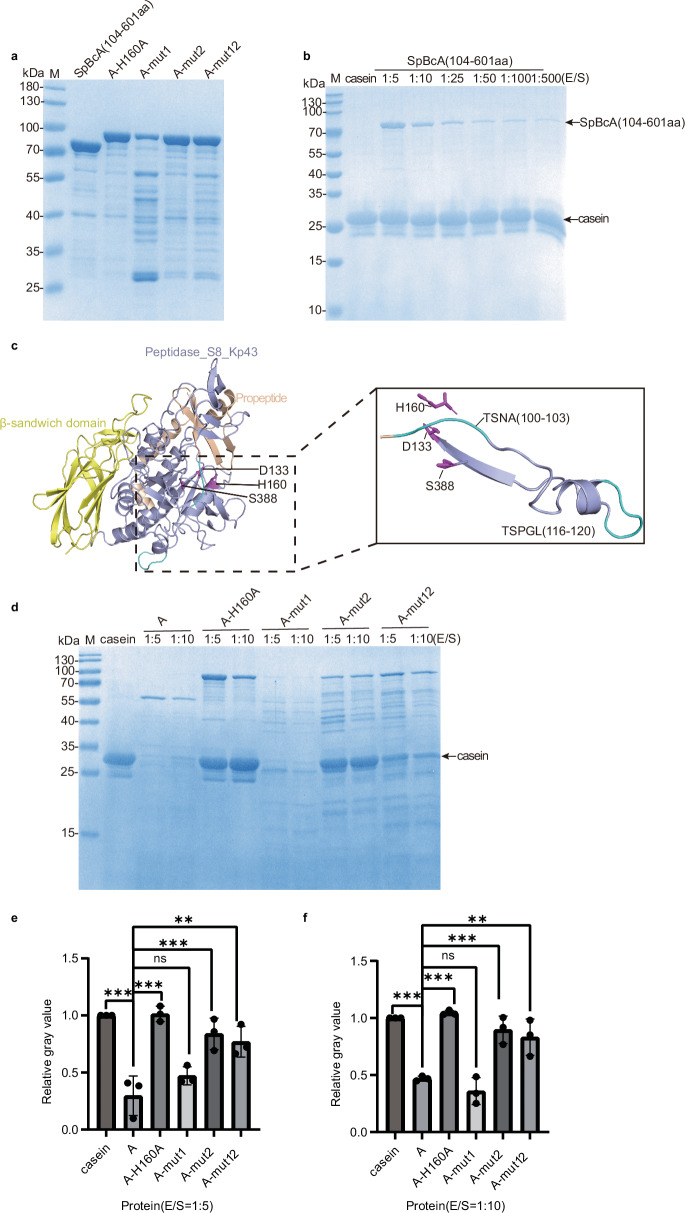
Purification and activity measurement of SpBcA (30–601 aa) mutants. **a** Analysis of purified SpBcA variants and mutants, including SpBcA (104–601 aa), A-H160A, A-mut1, A-mut2 and A-mut12, via SDS‒PAGE. **b** Degradation of β-casein by SpBcA (104–601 aa). β-casein (final concentration of 0.87 µg/µL) was mixed with different amounts of SpBcA (104‒601 aa) and incubated for 30 min. The final concentration ratios of SpBcA (Enzyme, E) to β-casein (Substrate, S) at were 1:5, 1:10, 1:25, 1:50, 1:100 and 1:500 (E/S, enzyme:substrate). **c** The cleavage sites of the N-terminal propeptide of A. The structure of A is colored in the same scheme as in Fig. 2. The cleavage sites are colored in cyan. **d** Degradation of β-casein by A, A-H160A, A-mut1, A-mut2, and A-mut12. β-casein (final concentration of 0.87 µg/µL) was mixed with different amounts of peptidases and incubated for 30 min. The final concentration ratios of A, A-H160A, A-mut1, A-mut2, and A-mut12 (Enzyme, E) to β-casein (Substrate, S) were 1:5 and 1:10 (E/S, enzyme:substrate). **e**,**f** Quantification of the β-casein bands in panel (**d**) after the image was converted to black and white (Supplementary Fig. 10a). The band intensities of proteins A, A-H160A, A-mut1, A-mut2, and A-mut12 were quantified and compared at enzyme-to-substrate final concentration ratios of 1:5 (**e**) or 1:10 (**f**). The vertical axis represents the relative gray value of the β-casein bands. The error bars indicate the standard deviations from three experiments (*n* = 3). The data are shown as the means ± SD. Statistical significance was assessed using one-way ANOVA: * (*p* < 0.05), ** (*p* < 0.01), and *** (*p* < 0.001).

To study the effect of the N-terminal domain on peptidase activity, we generated truncation variants lacking the N-terminal domain and tested their peptidase activity. To our surprise, when we deleted the N-terminal domain of the peptidase SpBcA (104–601 aa) (Fig. 8a), the peptidase was still inactive on casein (Fig. 8b), possibly because the N-terminal domain might be required for the correct folding of the protein. To further verify the inhibitory effect of the N-terminal domain, we blocked self-cleavage and checked whether blocking self-cleavage inhibited the protease activity of A.

To block self-cleavage, we searched for putative self-cleavage sites on A. On the basis of structure prediction, the peptide TSNA (100–103 aa) (Fig. 8c), which passes through the catalytic triad, was a potential self-cleavage site. To test this hypothesis, we performed N-terminal sequencing of the self-cleavage product of peptidase A with the Edman degradation method. The results revealed that the N-terminus of the self-cleavage product of A is Asn-Ala-Gly-Ala-Ala-Glu-Gly-Thr-Gly-Val (NAGAAEGTGV), starting at amino acid N102, which is surrounded by the catalytic triad D133-H160-S388, as predicted by the structure (Supplementary Fig. 8 and Supplementary Fig. 9). Therefore, we replaced TSNA with AAAA by site-directed mutagenesis (A-mut1, Fig. 8a) and tested its activity. Surprisingly, this mutant could still degrade casein (Fig. 8d).

As a previous paper reported two self-cleavage sites on the peptidase NbSLP1 and mutation of both sites was required to inhibit self-cleavage^36^, we also searched for additional potential cleavage sites on peptidase A. We suspected that residues 116–120 aa (TSPGL) (Fig. 8c) may be the extra cleavage site, as they also contain a “TS” sequence. To test our hypothesis, we generated two additional mutants: A-mut2, in which residues 116–120 aa (TSPGL) were mutated to AAAAA, and the double-site mutant A-mut12, in which both peptides 100–103 aa and 116–120 aa were mutated to alanines (Fig. 8a). Testing the protease activity on casein revealed that the activity of A-mut2 was significantly reduced, whereas the activity of A-mut12 appeared to be an intermediate of the activities of A-mut1 and A-mut2 (Fig. 8d). To facilitate interpretation, we converted the gel in Fig. 8d to black and white (Supplementary Fig. 10a), quantified the intensity of the casein bands by measuring the grayscale values, and compared the relative intensities of the bands. The intensity of the bands is inversely proportional to the extent of casein degradation; that is, weaker bands indicate stronger casein degradation by the protease, reflecting higher protease activity. The analysis further confirmed that at 1:5 (Fig. 8e) and 1:10 (Fig. 8f) protease-to-substrate ratios, wild-type A and A-mut1 presented the highest activity, A-mut12 was less active, and A-mut2 presented very weak activity, similar to the activity of the catalytic mutant. This finding implies that residues 116–120 aa (TSPGL) likely constitute one of the self-cleavage sites. In summary, we discovered an inhibitory domain that was not similar to any inhibitory domains reported in other proteases thus far and revealed both the self-cleavage sites and the self-inhibition mechanism of peptidase A.

### Analysis of the virulence potential of A in vitro and in vivo

**Fig. 9:**
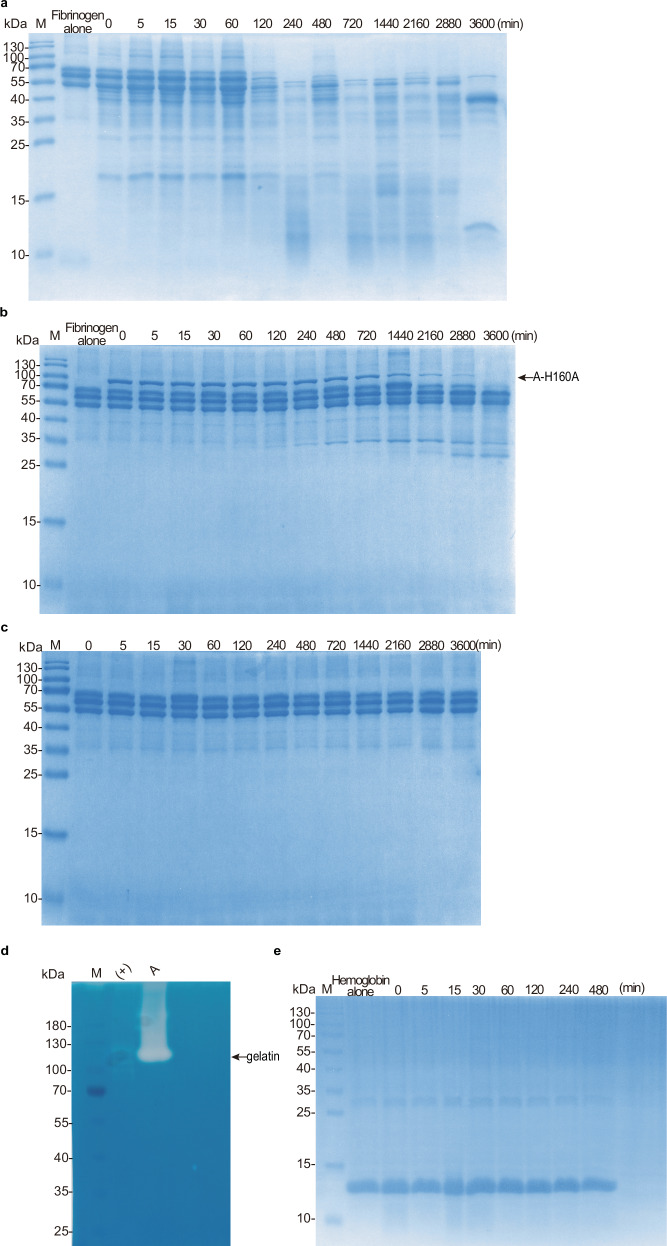
Fibrinogen and gelatin are substrates for the peptidase SpBcA (30–601 aa). Degradation of fibrinogen by A and A-H160A over time. Fibrinogen (final concentration of 1.91 µg/µL) was preincubated at a final concentration of 0.191 µg/µL peptidase A (**a**) or A-H160A (**b**) in a 115 μL reaction mixture containing 100 mM Tris-HCl, 150 mM NaCl, 5 mM CaCl_2_ and ddH_2_O, pH 7.4. At the indicated time points, aliquots (10 μL) were taken from the reaction mixture and processed as described in “Methods” section. **c** Fibrinogen (final concentration of 1.91 µg/µL) was used as a negative control in 115 μL of buffer, which consisted of 100 mM Tris-HCl, 150 mM NaCl, 5 mM CaCl_2_ and ddH_2_O, pH 7.4. **d** Degradation of gelatin by A. Gelatin was preincubated with peptidase A and the positive control (+) (collagenase IV) provided with the kit. **e** Degradation of hemoglobin by A. Hemoglobin (final concentration of 0.96 µg/µL) was preincubated with peptidase A (final concentration of 0.096 µg/µL) in 115 μL of 100 mM Tris-HCl, 150 mM NaCl, 5 mM CaCl_2_ and ddH_2_O, pH 7.4. At the indicated time points, aliquots (10 μL) were taken from the reaction mixture and processed as described in “Methods” section.

**Fig. 10:**
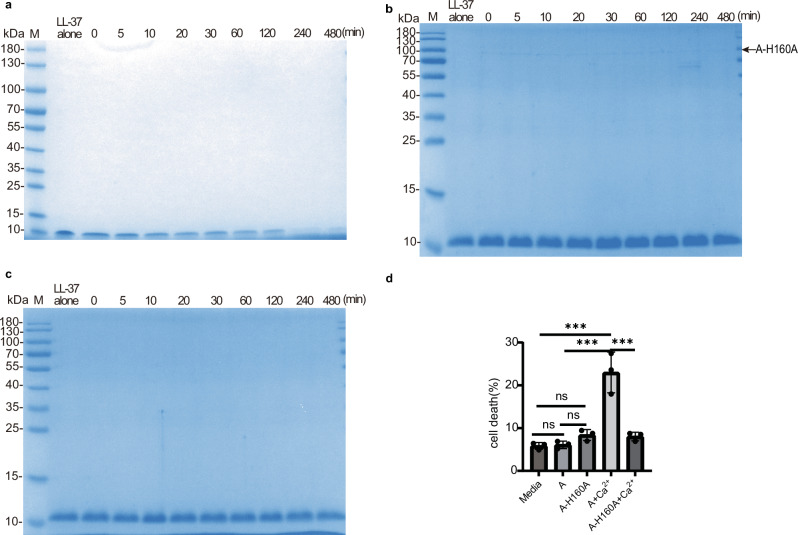
Evaluation of the virulence potential of the peptidase SpBcA (30–601 aa) in vitro and in vivo. **a**,**b** Degradation of the antimicrobial peptide LL-37 by A and A-H160A. The antimicrobial peptide LL-37 (final concentration of 0.17 µg/µL) was preincubated with (final concentration of 0.0017 µg/µL) peptidase A (**a**) or A-H160A (**b**) in 115 μL of 100 mM Tris-HCl, 150 mM NaCl, 5 mM CaCl_2_ and ddH_2_O, pH 7.4. At the indicated time points, aliquots (10 μL) were taken from the reaction mixture and processed as described in “Methods” section. **c** The antimicrobial peptide LL-37 (final concentration of 0.17 µg/µL) was used as a negative control in 115 μL of buffer, which consisted of 100 mM Tris-HCl, 150 mM NaCl, 5 mM CaCl_2_ and ddH_2_O, pH 7.4. **d** Analysis of cell death by LDH assays. HEK293T cells were transfected with A or A-H160A (10 μg) with or without Ca^2+^ and cell death was analyzed by LDH after 12 h (*n* = 3 biologically independent samples). The error bars indicate the standard deviations from three experiments (*n* = 3). The data are shown as the means ± SD. Statistical significance was assessed using one-way ANOVA: ns not significant, * (*p* < 0.05), ** (*p* < 0.01), *** (*p* < 0.001).

Since peptidase A is the most active of the three proteins, we tested whether A has the potential to be a virulence factor. We assessed its ability to degrade the human proteins hemoglobin, fibrinogen, and gelatin, which are key components of host cells, and LL-37, an antimicrobial peptide from the antimicrobial peptide family. As shown by the protease assays, peptidase A (preincubated at 37 °C for 20 min) significantly degraded fibrinogen (Fig. 9a), gelatin (Fig. 9d) and the antimicrobial peptide LL-37 (Fig. 10a). In contrast, hemoglobin was cleaved much less efficiently (Fig. 9e). As a control, A-H160A (preincubated at 37 °C for 20 min) did not degrade fibrinogen (Fig. 9b) or the antimicrobial peptide LL-37 (Fig. 10b), and there was also no degradation after the self-incubation of fibrinogen (Fig. 9c) or the antimicrobial peptide LL-37 (Fig. 10c), further confirming the activity and toxicity of A.

To further investigate the toxicity of the peptidases in vivo, we transfected peptidase A and its catalytic mutant A-H160A into HEK293T cells, cultured them for 12 h and then assessed cell death by lactate dehydrogenase (LDH) assays. LDH is a cytoplasmic enzyme that is released into the cell culture medium in response to cellular damage, making it a good correlate for the presence of damage and toxicity in cells (cell death). The results indicated that transfecting peptidase A together with 5 mM Ca^2+^ into the cells led to a marked increase in cell death (Fig. 10d). On the other hand, transfecting the catalytic mutant A-H160A together with Ca^2+^ or transfecting A or A-H160A in the absence of Ca^2+^ had no effect on cell death (Fig. 10d). This implies that peptidase A is a potential virulence factor of *B. cardium* and that its protease activity is required for virulence.

## Discussion

In this study, we identified three T9SS effector proteins, peptidases SpBcA, B and C and analyzed their peptidase functions. We showed that A and B are active peptidases that can self-cleave, and their self-cleavage increases their activity in vitro. Structural analysis of A and B revealed an N-terminal domain of which the C-terminal peptide passes through the substrate binding site of the peptidases. This N-terminal domain (propeptide) shares no sequence or structural similarity with the inhibitory I9 domain, which is a common propeptide in subtilisin peptidases. Mutagenesis and biochemical assays further confirmed the activation by self-cleavage and revealed potential cleavage sites. In summary, this study identified new peptidases as potential T9SS effector proteins in *B. cardium* and revealed the self-cleavage activation mechanism of protease A by a unique N-terminal propeptide. The activity of peptidase A toward host cell components implies potential for the medical application of the peptidase.

When we expressed SpBcA, B and C recombinantly, we noticed that their properties were different, despite the high sequence similarity of the S8 peptidase domains of these proteins. A and B are active, whereas SpBcC is inactive in vitro. Sequence alignment revealed that the active center is conserved not only among the three peptidases but also with subtilisin and other S8 peptidases^36,37^. A comparison of the structures of SpBcA, B, C and a subtilisin-like alkaline serine protease, KP-43, from *Bacillus sp*. KSM-KP43^34^ revealed that SpBcC lacks the propeptide as well as an α-helix following the β-sandwich domain (Supplementary Fig. 10b), which could explain the differences in activity. A is more active than B in vitro, and B is more difficult to express and purify, possibly because of greater hydrophobicity, toxicity or partial misfolding^38,39^. Notably, SpBcC also exists as a longer isoform in the database (WP_260392832.1). Whether the longer form of SpBcC is active remains to be investigated.

As A exhibited the highest protease activity in vitro, our study focused mainly on A. We discovered that A underwent self-cleavage during our assays and that self-cleavage activated its protease activity. How self-cleavage activates peptidase A is an intriguing question. On the basis of structure prediction, the domain located at the N-terminus of the S8 peptidase domain (30–103 aa) contains a peptide that passes through the active center of SpBcA. We suspected that this domain might act as an inhibitory propeptide for A and that self-cleavage would release this inhibition, as was observed in many subtilisin-like peptidases. For example, *Bacillus subtilis* protease is synthesized as a proenzyme, with a propeptide of approximately 77 residues located between the N-terminal signal peptide and the protease domain^40^. The *Bacillus subtilis* protease subsequently cleaves the propeptide through an intramolecular autocatalytic mechanism, resulting in the maturation of the proenzyme into the active protease^41^. This propeptide is also crucial for the folding of the catalytic domain into its native state^42,43^. The I9 domain is the most common propeptide found in subtilisin, and most members of the I9 family are not independent proteins^22,44^. However, the N-terminal propeptide of A differs from the known I9 domain in protein sequence and structure. These findings suggest that we have identified a new potential inhibitory domain that regulates the activity of A.

Indeed, the enzymatic activity of A increased after self-cleavage in our assays. However, deletion of the propeptide also resulted in an inactive protein (SpBcA (104–601 aa)) instead of activating the peptidase. The propeptide may be required for proper folding of A, as was observed in the case of subtilisin. We subsequently attempted to identify the self-cleavage site of the propeptide. Our N-terminal sequencing data indicate that the peptide TSNA (100–103 aa), which traverses the active site of SpBcA, is indeed the self-cleavage site. However, mutation of residues 100–103 aa did not block self-cleavage. It is puzzling that the protein was still active even after mutation of this region. We speculate that the mutation in the 100–103 aa region might disrupt the interaction between the N-terminal domain and the active site, potentially decreasing the level of inhibition observed. A reduction in binding affinity due to mutation of the propeptide was observed in a previous study of a *Bacillus subtilis* protease homolog^45^. In that study^45^, the authors showed that mutating the C-terminal residues of the propeptide of the *Tk-Bacillus subtilis* protease impaired the optimal binding of the peptide to the substrate binding site near the active site of the peptidase, decreasing the inhibition by the propeptide. This seems to be consistent with our observations. Inspired by a study on the *Bacillus subtilis*-like protease NbSLP1^36^, in which two self-cleavage sites were identified, we identified a second potential cleavage site, 116–120 aa (TSPGL). Mutation of this region resulted in reduced protease activity, suggesting that it may serve as another cleavage site. We also performed self-cleavage assays for protein B, although no significant self-cleavage bands were detected by SDS-PAGE analysis for protein B, as the structural similarity between B and A implies that B might also be activated by self-cleavage. The results indicated that the activity of B was significantly increased after “self-cleavage” (*p* < 0.001). The precise cleavage sites and the molecular basis for the potential self-cleavage of B require further investigation.

Characterization of peptidase A also revealed its cation preference, optimal temperature and activity toward other biological substrates. Our study revealed that the activity of A is stimulated by Ca^2+^, similar to that of other peptidases^46,47^. The optimal temperature for the activity of A is 60 °C, similar to that of a newly isolated recombinant *Bacillus*-like protease from the psychrophilic bacterium *Shewanella arctica*^48^, a new keratinase from *Bacillus* sp. BK111^49^, a cuticle-degrading enzyme from *Purpureocillium lilacinum* (Pl_SerPep)^50^ and a crude xylanase from *Humicola* sp. Ly01^51^. In terms of biological substrates, A can degrade azocasein, casein, gelatin, fibrinogen and the antimicrobial peptide LL-37, similar to peptidases from other species, such as *Tannerella forsythia*^47^, *Streptococcus suis*^52^ and *R. anatipestifer*^19,53^. Notably, gelatin and fibrinogen are important factors in the host’s defense against infection and are often targeted by pathogens. The antimicrobial peptide LL-37 is derived from a tissue protein consisting of 37 residues and exhibits antibacterial activity. More importantly, when it is transfected into cells along with Ca^2+^, peptidase A can lead to cell death in HEK293T cells. This implies that peptidase A is indeed a virulence factor and that its protease activity is required for virulence. These studies revealed the potential role of SpBcA in the pathogenesis of *B. cardium*.

Our study presents opportunities for further exploration. First, our proposed mechanism is based on predicted structures. Although structure prediction for single proteins has become very reliable, obtaining the crystal structures of the peptidases would still provide more insights into the regulatory mechanism involved. Second, while we characterized the activity of the minimally active domains of the peptidases in vitro, conducting studies in the presence of additional domains would enrich our understanding. Third, by establishing a genetic system in *B. cardium* and investigating the role of peptidases in the *B. cardium*-host interaction, we can gain deeper insights into their biological functions. Our study provides a solid foundation for future studies aimed at uncovering the mechanisms of virulence in *B. cardium*.

## Conclusion

In summary, we identified three subtilisin-like proteases, SpBcA, SpBcB and SpBcC, in *B. cardium* and analyzed their structure and function. We found that SpBcA is produced in the form of a zymogen and undergoes autocleavage. An inhibitory propeptide at the N-terminus functions as a molecular chaperone and can inhibit its activity. These peptidases are likely to play important roles as virulence factors in the pathogenicity of *B. cardium*. Our study provides a foundation for further exploration of the pathogenic mechanisms of *B. cardium*.

## Methods

### Bacterial strains, plasmids, cell lines and growth conditions

The bacterial strains and plasmids used in this study are listed in Supplementary Table 2 (Supplementary Table 2). *B. cardium* HPQL was cultured on Columbia blood agar plates (Thermo Fisher Scientific) for 72 h at 35 °C. *Escherichia coli* DH5α and BL21(DE3) were used for cloning and protein expression, respectively. *E. coli* bacterial strains were cultured at 37 °C in Luria–Bertani (LB) medium (Hopebio, #HB0128) with or without ampicillin (100 mg/mL) as needed. HEK293T cells were obtained from the Stem Cell Bank, Chinese Academy of Sciences (CAS), Beijing, China. The cells were cultured in DMEM medium (Gibco, #11995065) supplemented with 10% FBS (Gibco, #A5670701) and 1% Penicillin- Streptomycin-Amphotericin B Solution, and cultured in a 37 °C incubator with 5% CO_2_.

### Cloning and protein expression

The genes encoding SpBcA, B and C were identified by searching the National Center for Biotechnology Information (https://www.ncbi.nlm.nih.gov/) database, using the “*Bergeyella cardium* HPQL and S8 family of peptidases” as inputs, and the results are listed in Supplementary Table 1. Six proteins were identified. The sequences of QHN65150.1 and QHN65200.1 are identical; QHN65828.1 and QHN64674.1 contain only the S8 peptidase domain. Therefore, we chose the three proteins QHN65200.1 (SpBcA), QHN65156.1 (SpBcB), and QHN66091.1 (SpBcC), which contain both the S8 peptidase domain and the CTD domain. The full-length target genes were amplified using the genomic DNA of strain *B. cardium* HPQL as a PCR template and cloned and inserted into the BamHI (Takara, #1605) and XhoI (Takara, #1605) restriction sites of the pGEX-6p-2 vector, resulting in proteins with a GST tag, followed by the Precision protease cleavage site, at the N-terminus of the protein. The truncation variants were cloned in the same way, except that the plasmids containing the full-length genes were used as PCR templates. Catalytic mutants and inhibitory mutants of SpBcA were generated by site-directed mutagenesis PCR (Quik change site-directed mutagenesis). The primers used are listed in Supplementary Table 3 (Supplementary Table 3). Plasmid sequencing was carried out at Sangon Biotech (Shanghai) throughout the study.

The proteins were expressed in *E. coli* BL21(DE3) in LB media (Hopebio, #HB0128) supplemented with 100 mg/mL ampicillin (Solarbio, #A8180) overnight. Briefly, 0.6 L of *E. coli* BL21(DE3) transformed with the corresponding plasmid was cultured at 37 °C until the OD_600_ reached 0.6–0.8. Protein expression was induced with 0.3 mM IPTG (Sigma, #I6758) at 20°C. The cells were harvested 14 h postinduction via centrifugation at 4000 rpm for 15 min. The bacterial pellet was used for protein purification.

### Protein purification

All protein purification steps were performed at 4 °C unless otherwise stated. The bacterial pellet from 0.6 L of liquid culture was resuspended in 40 mL of GST Lysis Buffer (50 mM Tris-HCl, 500 mM NaCl, 10% glycerol (v/v) and 1 mM DTT, pH 7.4). The cells were lysed by sonication at a power of 300 W for 30 min, with a working time of 3 seconds and a pause of 7 seconds. The cell lysates were clarified by centrifugation (11000 rpm, 1 h, 4 °C) and filtered through a 0.22-μm syringe filter (Millipore, #SLGPR33RB). The filtered supernatant was incubated with GST-tag Purification Resin (Beyotime, #P2251) at 4 °C for 1 h with rotation. Then, the supernatant was removed, and the GST-tag Purification Resin was washed with GST Washing Buffer (50 mM Tris-HCl, 500 mM NaCl, 10% glycerol (v/v) and 1 mM DTT, pH 7.4) at least 3 times. Finally, the target protein bound to the GST-tag Purification Resin was eluted using GST Elution Buffer (50 mM Tris-HCl, 500 mM NaCl and 10 mM GSH, pH 8.0). The eluent was thawed on ice and concentrated to the desired concentration in a 15 mL, 10 kDa cutoff ultrafiltration centrifuge tube (Millipore, #UFC901008). The samples were then divided into aliquots, which were placed in 1.5 mL Eppendorf tubes, flash-frozen in liquid nitrogen, and stored at -80 °C. The purified protein was analyzed by SDS‒PAGE.

### Sequence alignment

Alignments of SpBcA, B and C protein sequences and peptidases from *R. anatipestifer*, *Porphyromonas gingivalis*, and *Bacillus* sp. species were performed in Jalview using the ‘Tcoffee with Defaults’ algorithm. The alignment was colored on the basis of an identity of 60%. A deeper color represents a higher percentage of identity.

### Prediction of structural models

Structural models of SpBcA, B and C were predicted via the trRosseta online server (https://yanglab.qd.sdu.edu.cn/trRosetta/). Briefly, the protein sequences of SpBcA, B and C were used as input sequences for structure prediction, which was performed with default parameter settings. trRosetta provided 5 models as results for each prediction. The highest ranked model of each prediction, model 1, was used for further analysis. The confidence of the overall structure prediction is reflected by the TM score. A TM score (0–1) above 0.5 usually indicates a model with correct topology. The confidence of the prediction at each residue is indicated by per-residue LDDT scores, ranging from 0–100, located at the B-factor column of the PDB file of the structure. Structural figures were made using PyMOL (Schroedinger).

### Mass spectrometry analysis

Protein samples were separated on 15% SDS‒PAGE gels and stained with Coomassie Brilliant Blue (Solarbio, #P1305). First, they were washed in ultrapure water for 15 min and submerged in 300 µL of 100 mM NH_4_HCO_3_/30% (v/v) acetonitrile (ACN) (Sigma, #360457) solution until they became transparent. The proteins were subsequently reduced with 10 mM dithiothreitol and then alkylated with 50 mM iodoacetamide in the dark. Proteins were digested with sequencing grade trypsin at 37 °C overnight. The peptides were desalted and concentrated using C18-based solid phase extraction prior to analysis.

For mass spectrometry analysis, solution A was a 0.1% (v/v) formic acid aqueous solution, and solution B was a 0.1% (v/v) formic acid (80%) (v/v) acetonitrile aqueous solution. After the chromatographic column was equilibrated with 95% (v/v) solution A, the peptides were separated on a C18 column (15 cm, 2 µm C18) at 300 nL/min with a gradient increasing from 5% Buffer B/95% Buffer A to 35% Buffer B/65% Buffer A over 50 min.

Mass spectrometers (Orbitrap eclipse, Thermo Scientific) were operated in data-dependent (DDA) positive ion mode. A full MS scan range of 375–1800 and Orbitrap resolution of 60,000 were used for all the samples. The AGC target and maximum injection times were set to 1E6 and 200 ms, respectively. Data-dependent acquisition mode was used to trigger precursor isolation and sequencing. Precursor ions were isolated with an m/z window of 1.6 and fragmented by high-energy dissociation with a collision energy of 30%. The cycle time was 3 s. To minimize repeated sequencing, dynamic exclusion with a duration of 45 s was utilized.

The uniprotkb_*bergeyella cardium* dataset was used to search the original mass spectrometry files against the corresponding database, resulting in the identification of proteins. Qualitative analysis of the data was conducted using Byonic (v2.7) software. Methionine oxidation was selected as a variable modification. Carbamidomethylation of cysteine residues was set as a fixed modification.

### N-terminal sequencing of the SpBcA self-cleavage product

N-terminal sequence analysis was performed by Biotech Pack Scientific Co., Ltd. (Beijing) with the Edman degradation method. Briefly, 0.35 µg/µL (final concentration) peptidase A was incubated in 115 µL of reaction buffer (100 mM Tris-HCl, pH 7.4; 150 mM NaCl; 5 mM CaCl_2_; ddH_2_O) at 37 °C for 20 min for self-cleavage. The product of the reaction was analyzed by SDS‒PAGE. The protein bands were transferred to a PVDF membrane and visualized by staining with Coomassie Brilliant Blue (Solarbio, #P1305). The band corresponding to peptidase A was cut from the membrane and placed in a reactor. The reactor was assembled and then positioned in the PPSQ-30 protein sequencer (SHIMADZU). The sequencer utilizes phenyl isothiocyanate (PITC) to couple with the N-terminal α-amino group of proteins under weakly alkaline conditions, forming phenylthiohydantoin (PTC-protein). Under acidic conditions, the first residue at the N-terminus is cleaved and converted into a more stable PTH amino acid, which is then subjected to high-performance liquid chromatography (HPLC) (SHIMADZU, #235-63951) for online analysis. This reaction is repeated for 10 cycles to determine the amino acid residues and their sequence at the N-terminus of the protein being analyzed.

### Reciprocal cleavage between peptidases A and B

To study the cleavage of B-H160A by peptidases A and B, peptidase A or B was used as the enzyme to digest the substrate B-H160A (final concentration of 0.26 µg/µL). The final concentrations of the peptidases (0.052, 0.026, 0.0104, 0.0052, 0.0026 and 0.00052 µg/µL) were incubated with the substrate in buffer (100 mM Tris-HCl, pH 7.4; 150 mM NaCl; 5 mM CaCl_2_; ddH_2_O) for a final total volume of 115 μL at 37 °C for 30 min. Twenty microliter samples were analyzed by 15% SDS‒PAGE, followed by Coomassie blue staining. All the experiments were repeated three times. The cleavage of A-H160A by peptidases A and B was performed as described above, with the exception that A-H160A was used as the substrate instead of B-H160A. The same procedures were used for the digestion of wild-type A by B and the digestion of wild-type B by A, with the exception that the substrate was substituted by wild-type A or B, respectively.

### Protease activity assay

Unless otherwise specified, in all the experiments, the proteins were stored at -80 °C and placed on ice shortly before use. The peptidase activity was tested via the use of multiple substrates, including azocasein (Sigma, #A2765), bovine β-casein (Millipore Sigma, #C6905), bovine plasma fibrinogen (MeilunBio, #MB5809), human antimicrobial peptide LL-37 (AnaSpec, #As-61302) and hemoglobin (Sigma, #H7379).

For assays using azocasein as a substrate, unless otherwise specified, the protease assays substrate were performed at 60 °C for 30 min in 80 µL of reaction mixture containing 2.5 µg/µL (final concentration) azocasein (Sigma, #A2765) and 0.05 µg/µL (final concentration) enzyme in buffer (100 mM Tris-HCl, pH 7.4; 150 mM NaCl; 5 mM CaCl_2_; ddH_2_O). The reaction was terminated by adding 20 µL of 20% (wt/vol) trichloroacetic acid (TCA) (Macklin, #T992763), which precipitated the proteins in the reaction. The reaction system was centrifuged at 2000 × *g* for 10 min at room temperature to pellet the protein precipitate. The supernatant contained the reaction products and was added to an equal volume of 0.5 mol/L NaOH solution. The absorbance of this final solution was measured at 450 nm using a microplate reader. The control was obtained by mixing 20 µL of 20% TCA (wt/vol), 80 µL of azocasein alone and 100 µL of 0.5 mol/L NaOH without adding the enzyme. The assays used to study the optimal pH, temperature, effect of cations and optimal Ca^2+^ concentration were performed in the same way as described above, with variations in the factors investigated (pH: 5–11; temperature: 20–90 °C; cations: 0, Ca^2+^, Mg²⁺, Mn²⁺, Zn²⁺, EDTA, or no ions; and a concentration gradient of CaCl_2_ (0 mM to 100 mM)).

For the titration assays in which azocasein was used as a substrate, as shown in Fig. 4e, f, the procedures were generally the same as those described above, with variations in the final concentration of the protease (0.5, 0.25, 0.1, 0.05, 0.025 and 0.005 µg/µL). For the time course experiments in Fig. 5c–f, the procedures were generally the same as those described above, except that the final concentration of the protease was 0.25 µg/µL instead of 0.05 µg/µL to obtain a better signal, and the samples were taken at time points of 5 min, 10 min, 15 min, 20 min, 30 min and 60 min, respectively. For self-cleavage, the samples were preincubated at 60 °C for 20 min before proteolysis was started by adding azocasein. For reactions without self-cleavage, peptidase A was stored at -80 °C until the addition of azocasein in parallel with the reaction with self-cleavage.

For assays using the antimicrobial peptide LL-37, hemoglobin, and fibrinogen as substrates, A or A-H160A was incubated with the substrate in reaction buffer (100 mM Tris-HCl, pH 7.4; 150 mM NaCl; 5 mM CaCl_2_; ddH_2_O) at a final total volume of 115 μL. The specific conditions were as follows: A or A-H160A (final concentration of 0.0017 µg/µL) were incubated with the antimicrobial peptide LL-37 (final concentration of 0.17 µg/µL), resulting in an enzyme:LL-37 concentration ratio of 1:100; A or A-H160A (final concentration of 0.096 µg/µL) was incubated with hemoglobin (final concentration of 0.96 µg/µL), resulting in an enzyme:hemoglobin concentration ratio of 1:10; and A or A-H160A (final concentration of 0.191 µg/µL) was incubated with fibrinogen (final concentration of 1.91 µg/µL), resulting in an enzyme:fibrinogen concentration ratio of 1:10. For the time course experiments, proteolysis was stopped at specific time points (LL-37: 0, 5, 10, 20, 30, 60, 120, 240 and 480 min; hemoglobin: 0, 5, 15, 30, 60, 120, 240 and 480 min; bovine plasma fibrinogen: 0, 5, 15, 30, 60, 120, 240, 480, 720, 1440, 2160 and 3600 min). Hemoglobin and fibrinogen samples (20 μL) were analyzed by 15% SDS‒PAGE, and the antimicrobial peptide LL-37 was analyzed via 4‒20% Precast Protein Plus Gel (YEASEN, #36270ES10), followed by Coomassie brilliant blue (Coomassie) staining. For the negative control of fibrinogen and antimicrobial peptide LL-37, 1.91 µg/µL fibrinogen and 0.17 µg/µL LL-37 were incubated at 37 °C in a final total volume of 115 μL buffer (100 mM Tris-HCl, pH 7.4; 150 mM NaCl; 5 mM CaCl_2_; ddH_2_O) without adding any enzymes and were analyzed by 15% SDS‒PAGE. All other treatments were performed as described above.

For the assays using bovine β-casein as the substrate, shown in Supplementary Fig. 6c, d, 0.87 µg/µL (final concentration) β-casein was incubated with the peptidase enzyme:substrate concentration ratios of 1:5, 1:10, 1:25, 1:50, 1:100 and 1:500 in a final total volume of 115 μL of reaction buffer (100 mM Tris-HCl, pH 7.4; 150 mM NaCl; 5 mM CaCl_2_; ddH_2_O) at 37 °C for 30 min. Samples (10 μL) were taken and mixed with 2.5 μL of 5×SDS loading buffer. The samples were then denatured via incubation at 100 °C for 10 min, subjected to 15% SDS‒PAGE (Epizyme Biotech, #PG114) and stained with Coomassie Brilliant Blue Staining Reagent (Solarbio, #P1305). For the time course experiments in Supplementary Fig. 7a and c, β-casein (final concentration of 0.87 µg/µL) was incubated with peptidase A or B (final concentration of 0.087 µg/µL) in a final volume of 115 μL of reaction buffer (100 mM Tris-HCl, pH 7.4; 150 mM NaCl; 5 mM CaCl_2_; ddH_2_O) at 37 °C. Samples (20 µL) were taken at 0, 5, 10, 15, 20, 30 and 60 min. The time course experiments in Supplementary Fig. 7b and d were performed as described above, except that peptidases were preincubated at 37 °C for 20 min. All the experiments were repeated three times.

### Transfection of recombinant proteins

Proteins were transfected into HEK293T cells using Xfect™ protein transfection reagent (TaKaRa, #631324) following the manufacturer’s instructions. Briefly, one day before transfection, HEK293T cells were seeded in a 12-well cell culture plate at a density of 1 × 10^6^ cells per well in DMEM-F12. Before the cells were transfected, the complete DMEM-F12 culture medium was removed from the 12-well plate using a sterile pipette with a sterile biosafety cabinet, and the cells were washed with serum-free Opti-MEM to eliminate residual serum. Then, the cells were cultured further by adding 500 μL of fresh Opti-MEM to a 12-well plate. Next, proteins were transfected into the cells according to the reagent manual, and after 12 h of incubation, the supernatants of the cultured cells were collected. One set of experiments included 7 wells: a well containing only media as the blank background, a well with nontransfected cells later used to set up the total cell death standard, a control well with nontransfected cells, a well transfected with wild-type A, a well transfected with A-H160A, a well transfected with wild-type A in buffer containing 5 mM CaCl_2_, and a well transfected with A-H160A in buffer containing 5 mM CaCl_2_. The transfection experiment was repeated three times for quantification of cell death with the LDH release assay.

### Lactate dehydrogenase (LDH) release assay

Lactate dehydrogenase activity was measured with a cytotoxicity kit (Promega, #G1781). Briefly, 50 μL of supernatant was transferred from the sample wells to a 96-well plate, followed by the addition of 50 μL of Working Solution. Mixing was performed thoroughly and carefully to prevent the formation of bubbles. The mixture was incubated at room temperature in the dark for 30 min, after which 50 μL of Stop Solution was added to terminate the reaction. The absorbance values of the samples were then measured at 490 nm using a microplate reader (Thermo Electron Corporation, #PC). Cytotoxicity (%) was calculated using the following formula: [(X-Z)/(Y-Z)] × 100%, where X refers to the absorbance value of the nontransfected control well and the four transfected wells, respectively; Y refers to the absorbance value of the well with total cell death; and Z refers to the absorbance value of the blank background wells.

### Gelatin enzyme spectrum assay

The gelatin enzyme spectrum assay was performed according to the manufacturer’s instructions (Real-Times (Beijing) Biotechnology Co. Ltd., #710278). Briefly, an SDS‒PAGE gel containing gelatin, the substrate for collagenase, was prepared. Then, peptidase A and the positive control (+) (collagenase IV) provided by the kit were loaded onto the SDS‒PAGE gel, and electrophoresis was performed. During electrophoresis, SDS bound reversibly to the proteases, disrupting hydrogen bonds and hydrophobic interactions, thus preventing them from degrading gelatin. After electrophoresis was completed, the gel was incubated with a reactivation solution to restore protease activity, allowing hydrolysis of the gelatin in the gel at their migration positions, followed by Coomassie Brilliant Blue staining. Since the SDS‒PAGE gel contained protein substrates, it was deeply stained, resulting in a dark background. However, at the positions of the protease bands, the substrate was degraded by the proteases and could not be stained with Coomassie Brilliant Blue, resulting in clear white areas, which allowed simultaneous determination of the size and activity of the proteases (zymography).

### Quantification of casein bands

The bands in Fig. 8d were quantified via ImageJ. First, the color image was converted to a black and white image using Photoshop (Supplementary Fig. 10a) and subsequently to an 8-bit image. The region of interest was selected to obtain the peak profile, and the area of the peak profile corresponded to the grayscale value of the protein band. The absolute gray values of the β-casein bands in the control (casein alone) and experimental groups were measured for each of the three independent replicates. The average absolute gray values were then divided by the average absolute gray value of the control β-casein bands. Therefore, the relative gray value of the control casein band was 1. When β-casein was degraded by proteases, the value was less than 1, inversely proportional to the enzyme activity.

### Statistics and reproducibility

In this study, statistical significance was calculated using the GraphPad Prism software (version 10.4.1). The significance was indicated by *p* values. Different letters or asterisks in the graphs indicate significant differences: ns, not significant, * (*p* < 0.05), ** (*p* < 0.01), *** (*p* < 0.001). The means ± SD represent the means and standard deviations calculated from three replicates. Statistical analysis was performed using one-way ANOVA (algorithm: Dunnett’s multiple comparison) (Fig. 3a, b, d; Fig. 4e, f; Fig. 8e, f; Fig. 10d) or two-way ANOVA (algorithm: Šidák multiple comparison) (Fig. 5c–f). The relationship between Ca^2+^ concentration and protein activity was assessed using the Spearman correlation coefficient (Fig. 3c). The specific methods used can be found in the figure legends. Differences between groups were considered significant for *p* values < 0.05. The enzyme assays were performed in triplicate, and the standard error of the mean is shown by vertical bars in the figures.

### Reporting summary

Further information on research design is available in the Nature Portfolio Reporting Summary linked to this article.

## Supplementary information

Supplementary Information

Description of Additional Supplementary Files

Supplementary Data 1

Supplementary Data 2

Supplementary Data 3

Supplementary Data 4

Supplementary Data 5

Supplementary Data 6

Supplementary Data 7

Supplementary Data 8

Supplementary Data 9

Supplementary Data 10

Supplementary Data 11

Supplementary Data 12

Reporting Summary

## Data Availability

The PDB code for the (Ser221Cys)-Subtilisin E-propeptide complex that was used in structural comparison is 1SCJ. If needed, the authors can provide the theoretical structures of SpBcA, SpBcB and SpBcC predicted by trRosetta. The Supplementary information document contains all Supplementary Figs. (Supplementary Fig. 1–10) and Supplementary Tables (Supplementary Table 1–3). Supplementary Data 1 contains statistical source data. Supplementary Data 2 contains the original uncropped gel images. Supplementary Data 3–7 provide the original N-terminal sequencing data corresponding to Supplementary Fig. 8, and Supplementary Data 8–12 provide the original N-terminal sequencing data corresponding to Supplementary Fig. 9. Materials generated in this study will be freely available to any researcher upon reasonable request.

## Code availability

No custom code or mathematical algorithm was used in this study.

## References

[1] Sohn, K.M. et al. A new causative bacteria of infective endocarditis, Bergeyella cardium sp. nov. *Diagn. Microbiol. Infect. Dis.* **81**, 213–216 (2015). 25544000 10.1016/j.diagmicrobio.2014.12.001

[2] Petrela, R.B., Lieberman, J.A. & Swan, R.T. An Unnamed Human Oral Bergeyella sp. as the Cause of an Unusual Bacterial Keratitis. *Case Rep. Ophthalmological Med.* **2023**, 3288984 (2023). 10.1155/2023/3288984 PMC10234703 37273837

[3] Liu, G. et al. Characterization and the first complete genome sequence of a novel strain of Bergeyella porcorum isolated from pigs in China. *BMC Microbiol.* **24**, 214 (2024). 38886642 10.1186/s12866-024-03366-6 PMC11181579

[4] Guo, L.N. et al. Microbiological characteristics of a novel species most closely related to ‘Bergeyella cardium’ as a pathogen of infectious endocarditis. *PloS one* **13**, e0191715 (2018). 29370239 10.1371/journal.pone.0191715 PMC5784969

[5] Grams, T.R., Kim, D.Y. & McElvania, E. The brief case: Bergeyella zoohelcum bacteremia in an immunocompromised 69-year-old patient. *J. Clin. Microbiol.* **61**, e0040822 (2023). 36951461 10.1128/jcm.00408-22 PMC10035328

[6] Chen, Y. et al. Bacteremia caused by Bergeyella zoohelcum in an infective endocarditis patient: case report and review of literature. *BMC Infect. Dis.* **17**, 271 (2017). 28403835 10.1186/s12879-017-2391-z PMC5389159

[7] Cai, F. et al. Systematic microbiome dysbiosis is associated with IgA nephropathy. *Microbiol. Spectr.* **11**, e0520222 (2023). 37227280 10.1128/spectrum.05202-22 PMC10269816

[8] Cao, Y. et al. Comparative analyses of subgingival microbiome in chronic periodontitis patients with and without IgA nephropathy by high throughput 16S rRNA sequencing. *Cell. Physiol. Biochem.* **47**, 774–783 (2018). 29807361 10.1159/000490029

[9] Jackson, H.T. et al. Culture-independent evaluation of the appendix and rectum microbiomes in children with and without appendicitis. *PloS one* **9**, e95414 (2014). 24759879 10.1371/journal.pone.0095414 PMC3997405

[10] Park, P.H. et al. Association between gut microbiota and CpG island methylator phenotype in colorectal cancer. *Gut microbes* **16**, 2363012 (2024). 38860458 10.1080/19490976.2024.2363012 PMC11174071

[11] Zhang, X., Li, C., Cao, W. & Zhang, Z. Alterations of gastric microbiota in gastric cancer and precancerous stages. *Front. Cell. Infect. Microbiol.* **11**, 559148 (2021). 33747975 10.3389/fcimb.2021.559148 PMC7966516

[12] Braun, T.F., Khubbar, M.K., Saffarini, D.A. & McBride, M.J. Flavobacterium johnsoniae gliding motility genes identified by mariner mutagenesis. *J. Bacteriol.* **187**, 6943–6952 (2005). 16199564 10.1128/JB.187.20.6943-6952.2005 PMC1251627

[13] Hunnicutt, D.W., Kempf, M.J. & McBride, M.J. Mutations in Flavobacterium johnsoniae gldF and gldG disrupt gliding motility and interfere with membrane localization of GldA. *J. Bacteriol.* **184**, 2370–2378 (2002). 11948149 10.1128/JB.184.9.2370-2378.2002 PMC134979

[14] Li, N. et al. The type IX secretion system is required for virulence of the fish pathogen flavobacterium columnare. *Appl. Environ. Microbiol.* **83**10.1128/aem.01769-17 (2017). 10.1128/AEM.01769-17 PMC5691404 28939608

[15] Veillard, F. et al. Proteolytic processing and activation of gingipain zymogens secreted by T9SS of Porphyromonas gingivalis. *Biochimie* **166**, 161–172 (2019). 31212040 10.1016/j.biochi.2019.06.010 PMC6815250

[16] Ingmer, H. & Brøndsted, L. Proteases in bacterial pathogenesis. *Res. Microbiol.* **160**, 704–710 (2009). 19778606 10.1016/j.resmic.2009.08.017

[17] Koziel, J. & Potempa, J. Protease-armed bacteria in the skin. *Cell tissue Res.* **351**, 325–337 (2013). 22358849 10.1007/s00441-012-1355-2 PMC3560952

[18] Palm, E., Khalaf, H. & Bengtsson, T. Suppression of inflammatory responses of human gingival fibroblasts by gingipains from Porphyromonas gingivalis. *Mol. oral. Microbiol.* **30**, 74–85 (2015). 25055828 10.1111/omi.12073

[19] Chen, Z. et al. Riemerella anatipestifer T9SS effector SspA functions in bacterial virulence and defending natural host immunity. *Appl. Environ. Microbiol.* **88**, e0240921 (2022). 35575548 10.1128/aem.02409-21 PMC9195944

[20] Ehrmann, M. & Clausen, T. Proteolysis as a regulatory mechanism. *Annu. Rev. Genet.* **38**, 709–724 (2004). 15568990 10.1146/annurev.genet.38.072902.093416

[21] Shinde, U. & Thomas, G. Insights from bacterial subtilases into the mechanisms of intramolecular chaperone-mediated activation of furin. *Methods Mol. Biol. (Clifton, N. J.)* **768**, 59–106 (2011). 10.1007/978-1-61779-204-5_4 PMC4300103 21805238

[22] Hohl, M., Stintzi, A. & Schaller, A. A novel subtilase inhibitor in plants shows structural and functional similarities to protease propeptides. *J. Biol. Chem.* **292**, 6389–6401 (2017). 28223360 10.1074/jbc.M117.775445 PMC5391766

[23] Liu, X. et al. The Subtilisin-Like Protease Bcser2 Affects the Sclerotial Formation, Conidiation and Virulence of Botrytis cinerea. *Int. J. Mol. Sci.* **21**10.3390/ijms21020603 (2020). 10.3390/ijms21020603 PMC7013506 31963451

[24] Yabuta, Y., Takagi, H., Inouye, M. & Shinde, U. Folding pathway mediated by an intramolecular chaperone: propeptide release modulates activation precision of pro-subtilisin. *J. Biol. Chem.* **276**, 44427–44434 (2001). 11577106 10.1074/jbc.M107573200

[25] Pan, H., Li, W., Sun, E. & Zhang, Y. Characterization and whole genome sequencing of a novel strain of Bergeyella cardium related to infective endocarditis. *BMC Microbiol.* **20**, (2020). 32. 32050896 10.1186/s12866-020-1715-0 PMC7017618

[26] Tomida, J. et al. Spodiobacter cordis gen. nov. sp. nov., a member of the family Flavobacteriaceae isolated from patients with infective endocarditis. *Microbiol. Immunol.* **63**, 111–118 (2019). 30817020 10.1111/1348-0421.12673

[27] Siezen, R.J. & Leunissen, J.A. Subtilases: The superfamily of subtilisin-like serine proteases. *Protein Sci.* **6**, 501–523 (1997). 9070434 10.1002/pro.5560060301 PMC2143677

[28] Veith, P.D. et al. Protein substrates of a novel secretion system are numerous in the Bacteroidetes phylum and have in common a cleavable C-terminal secretion signal, extensive post-translational modification, and cell-surface attachment. *J. Proteome Res.* **12**, 4449–4461 (2013). 24007199 10.1021/pr400487b

[29] Jing, H. et al. Archaeal surface layer proteins contain beta propeller, PKD, and beta helix domains and are related to metazoan cell surface proteins. *Struct. (Lond., Engl. : 1993)* **10**, 1453–1464 (2002). 10.1016/s0969-2126(02)00840-7 12377130

[30] Burn, T.C. et al. Analysis of the genomic sequence for the autosomal dominant polycystic kidney disease (PKD1) gene predicts the presence of a leucine-rich repeat. The American PKD1 Consortium (APKD1 Consortium). *Hum. Mol. Genet.* **4**, 575–582 (1995). 7633406 10.1093/hmg/4.4.575

[31] Pavloff, N. et al. Molecular cloning and structural characterization of the Arg-gingipain proteinase of Porphyromonas gingivalis. Biosynthesis as a proteinase-adhesin polyprotein. *J. Biol. Chem.* **270**, 1007–1010 (1995). 7836351 10.1074/jbc.270.3.1007

[32] Li, N. et al. Structure determination and analysis of a haemolytic gingipain adhesin domain from Porphyromonas gingivalis. *Mol. Microbiol.* **76**, 861–873 (2010). 20233299 10.1111/j.1365-2958.2010.07123.x

[33] de Diego, I. et al. The outer-membrane export signal of Porphyromonas gingivalis type IX secretion system (T9SS) is a conserved C-terminal β-sandwich domain. *Sci. Rep.* **6**, 23123 (2016). 27005013 10.1038/srep23123 PMC4804311

[34] Nonaka, T. et al. The crystal structure of an oxidatively stable subtilisin-like alkaline serine protease, KP-43, with a C-terminal beta-barrel domain. *J. Biol. Chem.* **279**, 47344–47351 (2004). 15342641 10.1074/jbc.M409089200

[35] Jain, S.C., Shinde, U., Li, Y., Inouye, M. & Berman, H.M. The crystal structure of an autoprocessed Ser221Cys-subtilisin E-propeptide complex at 2.0 A resolution. *J. Mol. Biol.* **284**, 137–144 (1998). 9811547 10.1006/jmbi.1998.2161

[36] Wang, R. et al. Maturation of subtilisin-like protease NbSLP1 from microsporidia Nosema bombycis. *Front. Cell. Infect. Microbiol.* **12**, 897509 (2022). 36046739 10.3389/fcimb.2022.897509 PMC9421246

[37] Muhammed, N.S. et al. Recombinant Production and Characterization of an Extracellular Subtilisin-Like Serine Protease from Acinetobacter baumannii of Fermented Food Origin. *protein J.* **40**, 419–435 (2021). 33870461 10.1007/s10930-021-09986-5 PMC8053418

[38] Beygmoradi, A., Homaei, A., Hemmati, R. & Fernandes, P. Recombinant protein expression: Challenges in production and folding related matters. *Int. J. Biol. Macromolecules* **233**, 123407 (2023). 10.1016/j.ijbiomac.2023.123407 36708896

[39] Kaur, J., Kumar, A. & Kaur, J. Strategies for optimization of heterologous protein expression in E. coli: Roadblocks and reinforcements. *Int. J. Biol. Macromolecules* **106**, 803–822 (2018). 10.1016/j.ijbiomac.2017.08.080 28830778

[40] Ikemura, H., Takagi, H. & Inouye, M. Requirement of pro-sequence for the production of active subtilisin E in Escherichia coli. *J. Biol. Chem.* **262**, 7859-7864 (1987). 3108260

[41] Ohta, Y. & Inouye, M. Pro-subtilisin E: purification and characterization of its autoprocessing to active subtilisin E in vitro. *Mol. microbiol*. **4**, 295–304 (1990). 10.1111/j.1365-2958.1990.tb00596.x 2110997

[42] Ikemura, H. & Inouye, M. In vitro processing of pro-subtilisin produced in Escherichia coli. *J. Biol. Chem.* **263**, 12959–12963 (1988). 3047114

[43] Zhu, X.L., Ohta, Y., Jordan, F. & Inouye, M. Pro-sequence of subtilisin can guide the refolding of denatured subtilisin in an intermolecular process. *Nature* **339**, 483–484 (1989). 2657436 10.1038/339483a0

[44] Schaller, A., Stintzi, A. & Graff, L. Subtilases - versatile tools for protein turnover, plant development, and interactions with the environment. *Physiologia Plant.* **145**, 52–66 (2012). 10.1111/j.1399-3054.2011.01529.x 21988125

[45] Uehara, R., Ueda, Y., You, D.J., Koga, Y. & Kanaya, S. Accelerated maturation of Tk-subtilisin by a Leu→Pro mutation at the C-terminus of the propeptide, which reduces the binding of the propeptide to Tk-subtilisin. *FEBS J.* **280**, 994–1006 (2013). 23237738 10.1111/febs.12091

[46] Foophow, T. et al. Crystal structure of a subtilisin homologue, Tk-SP, from Thermococcus kodakaraensis: requirement of a C-terminal beta-jelly roll domain for hyperstability. *J. Mol. Biol.* **400**, 865–877 (2010). 20595040 10.1016/j.jmb.2010.05.064

[47] Ksiazek, M. et al. Mirolase, a novel subtilisin-like serine protease from the periodontopathogen Tannerella forsythia. *Biol. Chem.* **396**, 261–275 (2015). 25391881 10.1515/hsz-2014-0256 PMC4682893

[48] Qoura, F., Kassab, E., Reisse, S., Antranikian, G. & Brueck, T. Characterization of a new, recombinant thermo-active subtilisin-like serine protease derived from Shewanella arctica %J Journal of molecular catalysis, B. *Enzymatic %J.* **116**, 16–23 (2015).

[49] Moridshahi, R., Bahreini, M., Sharifmoghaddam, M. & Asoodeh, A. Biochemical characterization of an alkaline surfactant-stable keratinase from a new keratinase producer, Bacillus zhangzhouensis. *Extremophiles: Life Extrem. Cond.* **24**, 693–704 (2020). 10.1007/s00792-020-01187-9 32617734

[50] Pedezzi, R. et al. Biochemical and biophysical properties of a recombinant serine peptidase from Purpureocillium lilacinum. *Biophys. Chem.* **296**, 106978 (2023). 36827753 10.1016/j.bpc.2023.106978

[51] Zhou, J. et al. Production and characterization of ethanol- and protease-tolerant and xylooligosaccharides-producing endoxylanase from Humicola sp. Ly01. *J. Microbiol. Biotechnol.* **23**, 794–801 (2013). 23676918 10.4014/jmb.1211.11003

[52] Bonifait, L. & Grenier, D. The SspA subtilisin-like protease of Streptococcus suis triggers a pro-inflammatory response in macrophages through a non-proteolytic mechanism. *BMC Microbiol.* **11**, 47 (2011). 21362190 10.1186/1471-2180-11-47 PMC3058005

[53] Bonifait, L. et al. The cell envelope subtilisin-like proteinase is a virulence determinant for Streptococcus suis. *BMC Microbiol.* **10**, 42 (2010). 20146817 10.1186/1471-2180-10-42 PMC2832634

