# Supplementary material for: Functional study of Bergeyella cardium KP-43 subfamily peptidases as putative T9SS cargo
Source: Commun Biol. 2025 Apr 9;8:586. doi: 10.1038/s42003-025-07996-y (PMC11982257; doi:10.1038/s42003-025-07996-y)
Supplement: Supplementary file 1 — Supplementary Information [file 42003_2025_7996_MOESM1_ESM.pdf]

Supplementary Fig. 1

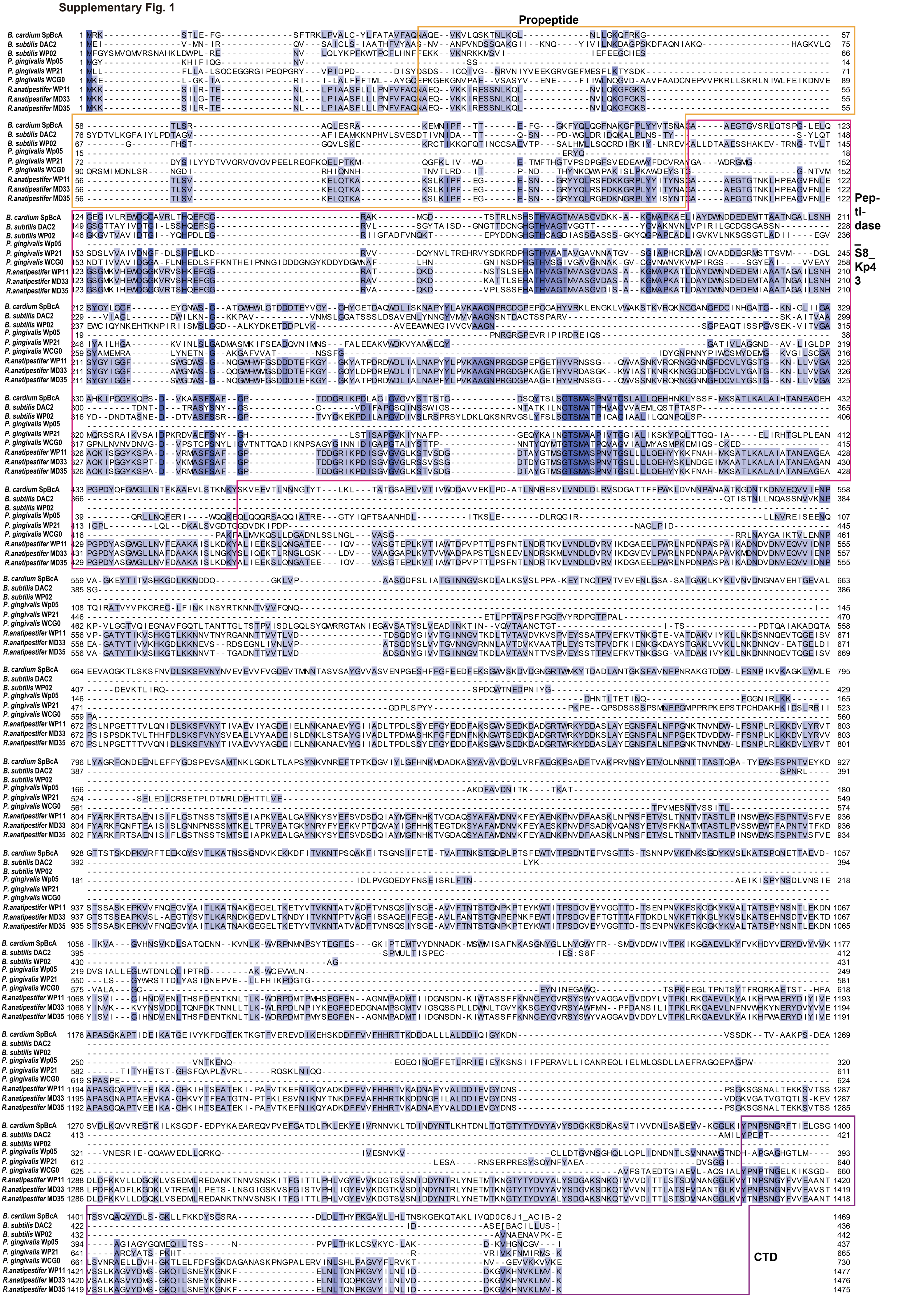

Supplementary Fig. 1

Multiple sequences alignment of the proteins from different species by Jalview

The names in the figure are all abbreviations, and the specific names are as follows. The full name of *B. cardium* is *Bergeyella cardium*; The full name of *B. subtilis* DAC2 is *Bacillus* D0C6J1_ACIB2; The full name of *B. subtilis* WP02 is *Bacillus* WP_024085327.1; The full name of *P. gingivalis* WP05 is *Porphyromonas gingivalis* WP_054191279.1; The full name of *P. gingivalis* WP21 is *Porphyromonas gingivalis* WP_211605210.1; The full name of *P. gingivalis* WCG0 is *Porphyromonas gingivalis* WCG03872.1; The full name of *R.anatipestifer* WP11 is *Riemerella anatipestifer* WP_112320221.1; The full name of *R.anatipestifer* MD33 is *Riemerella anatipestifer* MDY3343932.1; The full name of *R.anatipestifer* MD35 is *Riemerella anatipestifer* MDY3537089.1. The percentage of identity threshold for coloring is 60%. The deeper the color, the higher the accuracy of the sequence alignment.

Supplementary Fig. 2

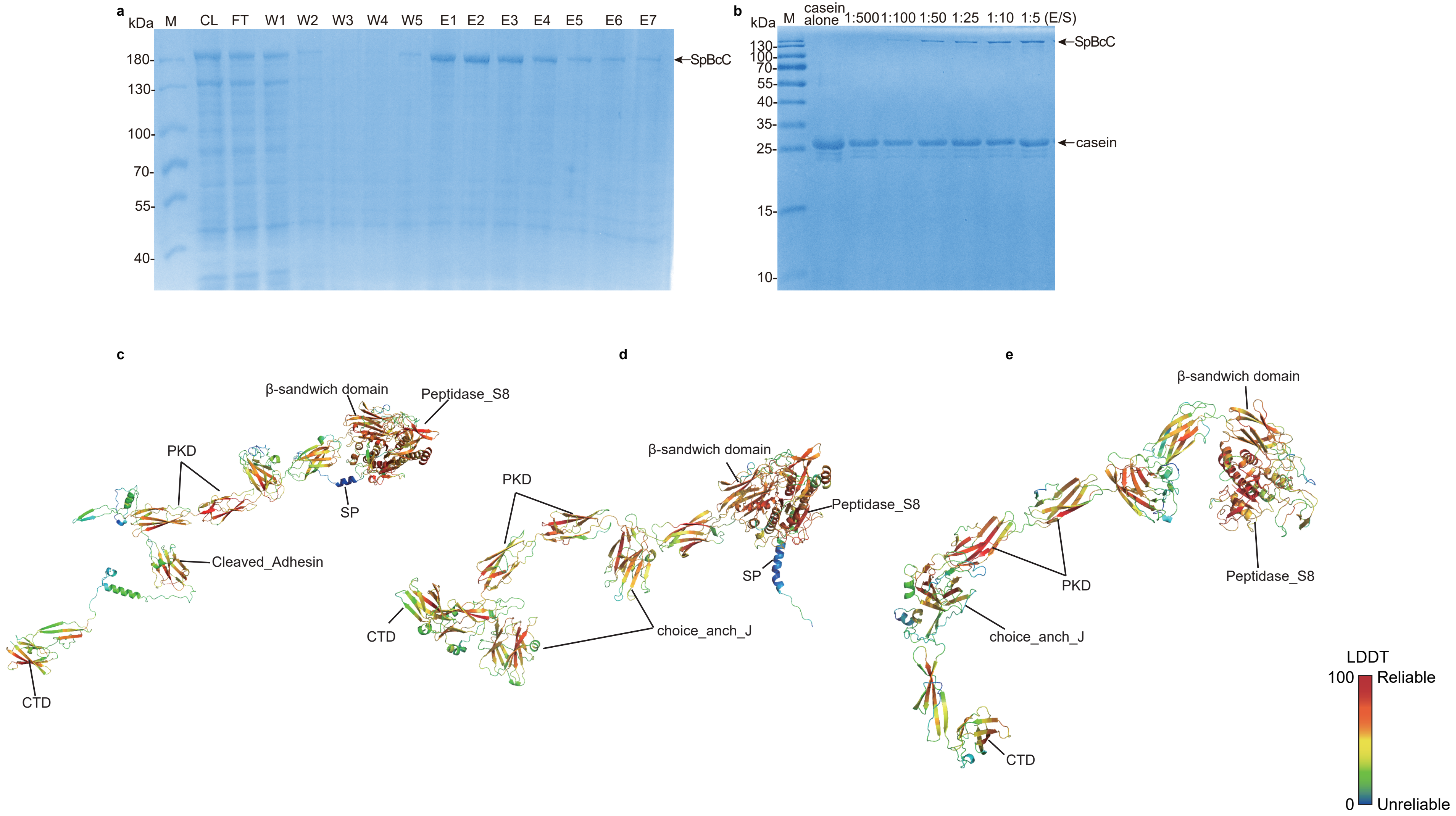

Supplementary Fig. 2

(a) Purification of SpBcC. CL: cell lysate, FT: flow through, W1-W5: wash fractions, E1-E7: elution fractions.

(b) Degradation of β-casein by SpBcC. β-casein (final concentration 0.87 μg/μL) was mixed with different amounts of SpBcC in 115 μL buffer of 100 mM Tris-HCl, pH 7.4, 150 mM NaCl and 5 mM $CaCl_2$ and incubated for 20 min. The final concentration ratio of SpBcC (Enzyme, E) to β-casein (Substrate, S) were 1:5, 1:10, 1:25, 1:50, 1:100 and 1:500 (E/S, enzyme/substrate).

(c)-(e) The predicted structures of SpBcA (c), SpBcB (d) and SpBcC (e). The confidence of the overall structure prediction is reflected by the TM-score. A TM-score (0-1) above 0.5 usually indicates a model with correct topology. The confidence of the prediction at each residue is indicated by per-residue LDDT scores, ranging from 0-100, locating at the B-factor column of the PDB file of the structure. The structures are colored based on the LDDT scores. Structural figures were made using PyMOL (Schroedinger).

Supplementary Fig. 3

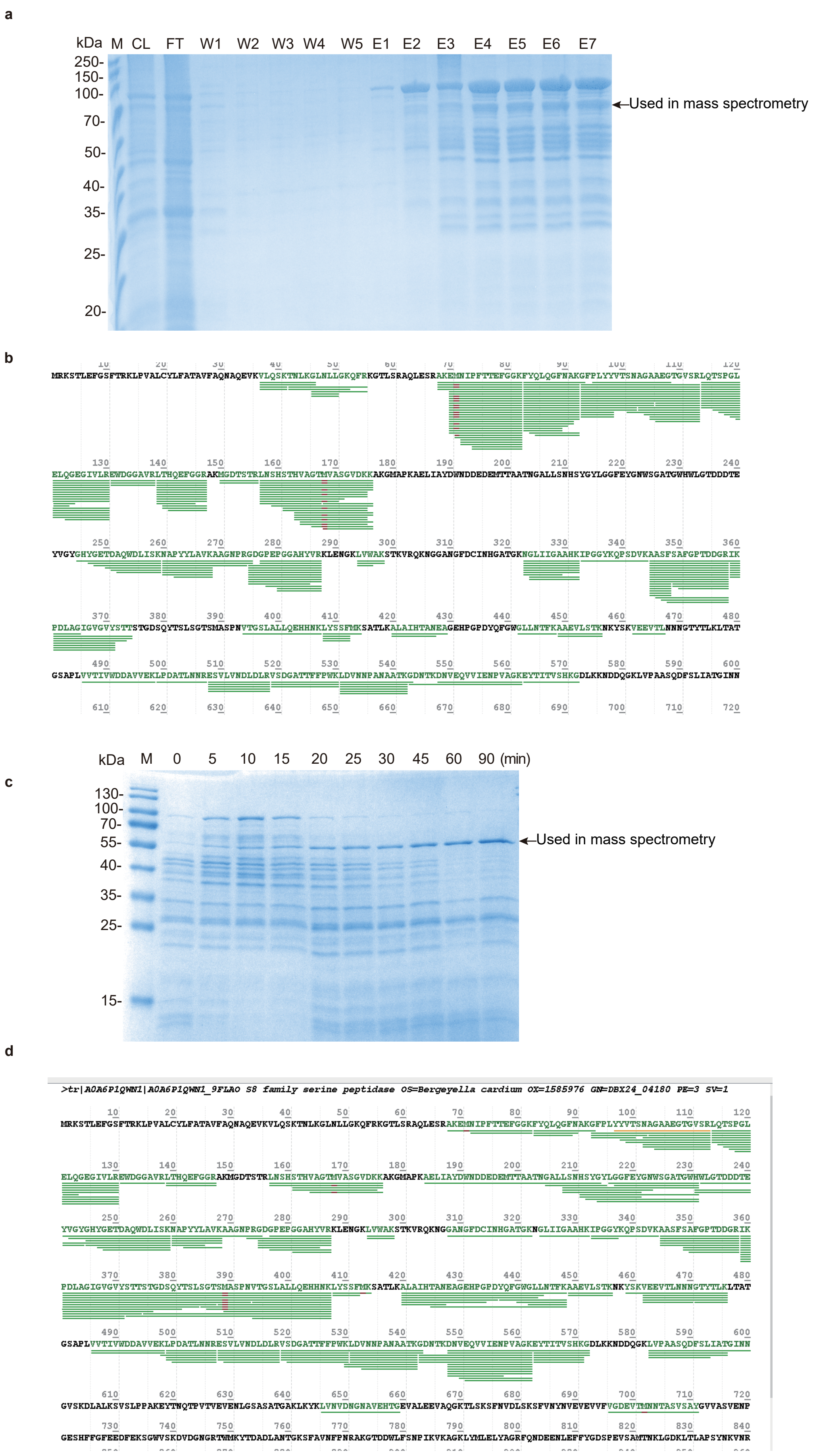

Supplementary Fig. 3

(a) and (b) Identification of protein bands during purification of A by mass spectrometry. The horizontal arrow, “→”, indicates the protein band used in the mass spectrometry experiment. Green bars indicate identified peptides, while red bars signify the presence of artificial modifications on amino acids, such as M representing oxidized methionine.

(c) and (d) Validation of the self-cleavage product of A by mass spectrometry experiment. The horizontal arrow, “→”, indicates the protein band used in the mass spectrometry experiment. Green bars indicate identified peptides, while red bars signify the presence of artificial modifications on amino acids, such as M representing oxidized methionine.

Supplementary Fig. 4

a

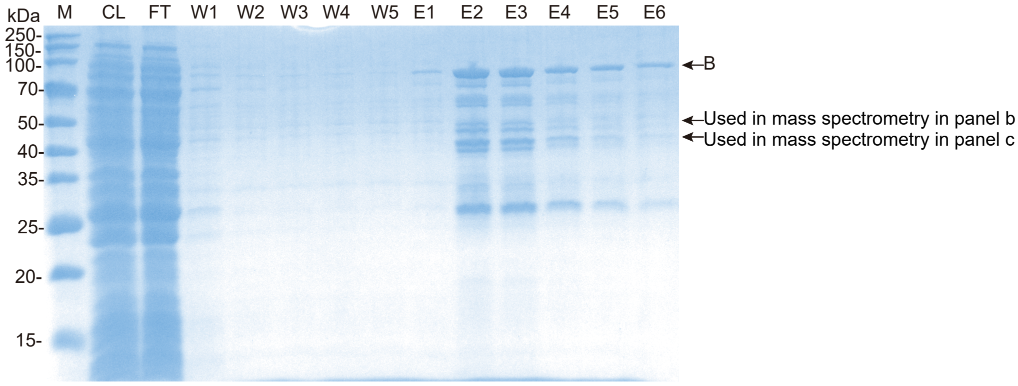

b

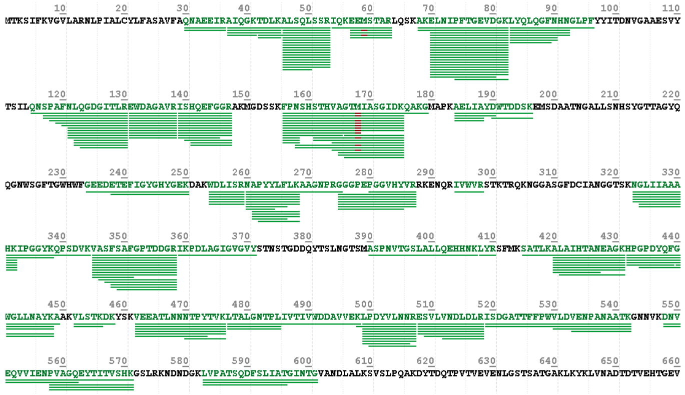

c

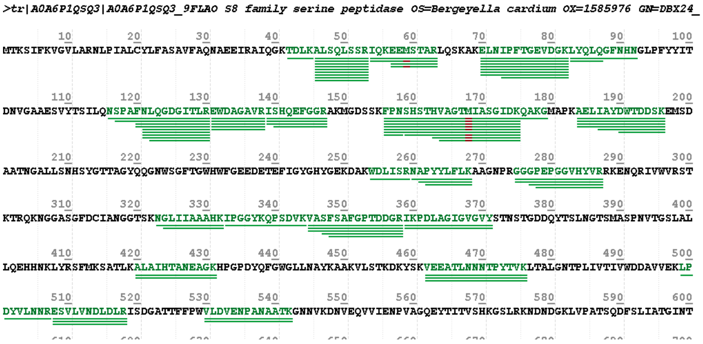

Supplementary Fig. 4

(a) Identification of protein bands during purification of B by mass spectrometry.

(b) and (c) The horizontal arrow, “→”, indicates the protein band used in the mass spectrometry experiment. Green bars indicate identified peptides, while red bars signify the presence of artificial modifications on amino acids, such as M representing oxidized methionine.

Supplementary Fig. 5

a

>sp|P0A6F5|CH60_ECOLI Chaperonin GroEL OS=Escherichia coli (strain K12) OX=83333 GN=groEL PE=1 SV=2

```
10 20 30 40 50 60 70 80 90 100 110 120
MAAKDVKFGNDARVKMLRGVNVLADAVKVTLGPKGRNVVLDKSFGAPTITKDGVSVAREIELEDKFENMGAQMVKEVASKANDAAGDGTTTATVLAQAIITEGLKAVAAGMNPMDLKRGI
130 140 150 160 170 180 190 200 210 220 230 240
DKAVTAAVEELKALSVPCSDSKAIAQVGTISANSDETVGKLIAEAMDKVGKEGVITVEDGTGLQDELDVVEGMQFDRGYLSPYFINKPETGAVELESPFILLADKKISNIREMLPVLEAV
250 260 270 280 290 300 310 320 330 340 350 360
AKAGKPLLIIAEDVEGEALATLVVNTMRGIVKVAAVKAPGFGDRRKAMLQDIATLTGGTVISEEIGMELEKATLEDLGQAKRVVINKDTTTIIDGVGEEAAIQGRVAQIRQQIEEATSDY
370 380 390 400 410 420 430 440 450 460 470 480
DREKLQERVAKLAGGVAVIKVGAATEVEMKEKKARVEDALHATRAAVEEGVVAGGGVALIRVASKLADLRGQNEDQNVGIKVALRAMEAPLRQIVLNCGEEPSVVANTVKGGDGNYGYNA
490 500 510 520 530 540
ATEEYGNMIDMGILDPTKVTRSALQYAASVAGLMITTECMVTDLPKNDAADLGAAGGMGGMGGMGGMM
```

b

>sp|P0A6H5|HSLU_ECOLI ATP-dependent protease ATPase subunit HslU OS=Escherichia coli (strain K12) OX=83333 GN=hslU PE=1 SV=1

```
10 20 30 40 50 60 70 80 90 100 110 120
MSEMTPREIVSELDKHIIGQDNAKRSVAIALRNRWRRMQLNEELRHEVTPKNILMIGPTGVGKTEIARRLAKLANAPFIKVEATKFTEVGYVGKEVDSIIRDLTDAAVKMVRVQAIEKNR
130 140 150 160 170 180 190 200 210 220 230 240
YRAEELAEERILDVLIPPAKNNWGQTEQQQEPSAARQAFRKKLREGQLDDKEIEIDLAAAPMGVEIMAPPGMEEMTSQLQSMFQNLGGQKQKARKLKIKDAMKLLIEEEAAKLVNPEELK
250 260 270 280 290 300 310 320 330 340 350 360
QDAIDAVEQHGIVFIDEIDKICKRGESSGPDVSREGVQRDLLPLVEGCTVSTKHGMVKTDHILFIASGAFQIAKPSDLIPELQGRLPIRVELQALTTSDFERILTEPNASITVQYKALMA
370 380 390 400 410 420 430 440
TEGVNIEFTDSGIKRIAEAAWQVNESTENIGARRLHTVLERLMEEISYDASDLSGQNITIDADYVSKHLDALVADEDLSRFIL
```

c

>sp|P0CE47|EFTU1_ECOLI Elongation factor Tu 1 OS=Escherichia coli (strain K12) OX=83333 GN=tufA PE=1 SV=1

```
10 20 30 40 50 60 70 80 90 100 110 120
MSKEKFERTKPHVNVGTIGHVDHGKTTLTAAITTVLAKTYGGAARAFDQIDNAPEEKARGITINTSHVEYDTPTRHYAHVDCPGHADYVKNMITGAAQMDGAILVVAATDGPMPQTREHI
130 140 150 160 170 180 190 200 210 220 230 240
LLGRQVGVPYIIVFLNKCDMVDDEELLELVEMEVRELLSQYDFPGDDTPIVRGSALKALEGDAEWEAKILELAGFLDSYIPEPERAIDKPFLLPIEDVFSISGRGTVVTGRVERGIIKVG
250 260 270 280 290 300 310 320 330 340 350 360
EEVEIVGIKETQKSTCTGVEMFRKLLDEGRAGENVGVLLRGIKREEIERGQVLAKPGTIKPHTKFESEVYILSKDEGGRHTPFFKGYRPQFYFRTTDVTGTIELPEGVEMVMPGDNIKMV
370 380 390
VTLIHPIAMDDGLRFAIREGGRTVGAGVVAKVLG
```

d

>sp|P02666|CASB_BOVIN Beta-casein OS=Bos taurus OX=9913 GN=CSN2 PE=1 SV=2

```
10 20 30 40 50 60 70 80 90 100 110 120
MKVLILACLVALALARELEELNVPGEIVESLSSSEESITRINKKIEKFQSEEQQQTEDELQDKIHPFAQTQSLVYPFPGPIPNSLPQNIPPLTQTPVVVPPFLQPEVMGVSKVKEAMAPK
130 140 150 160 170 180 190 200 210 220
HKEMPFPKYPVEPFTESQSLTLTDVENLHLPLPLLQSWMHQPHQPLPPTVMFPPQSVLSLSQSKVLPVPQKAVPYPQRDMPIQAFLLYQEPVLGPVRGPFPIIV
```

Supplementary Fig. 5

(a) - (d) Comparison of the mass spectrum results of peptidase B with the database of *Escherichia coli* proteins. Green bars indicate identified peptides, while red bars signify the presence of artificial modifications on amino acids, such as M representing oxidized methionine.

Supplementary Fig. 6

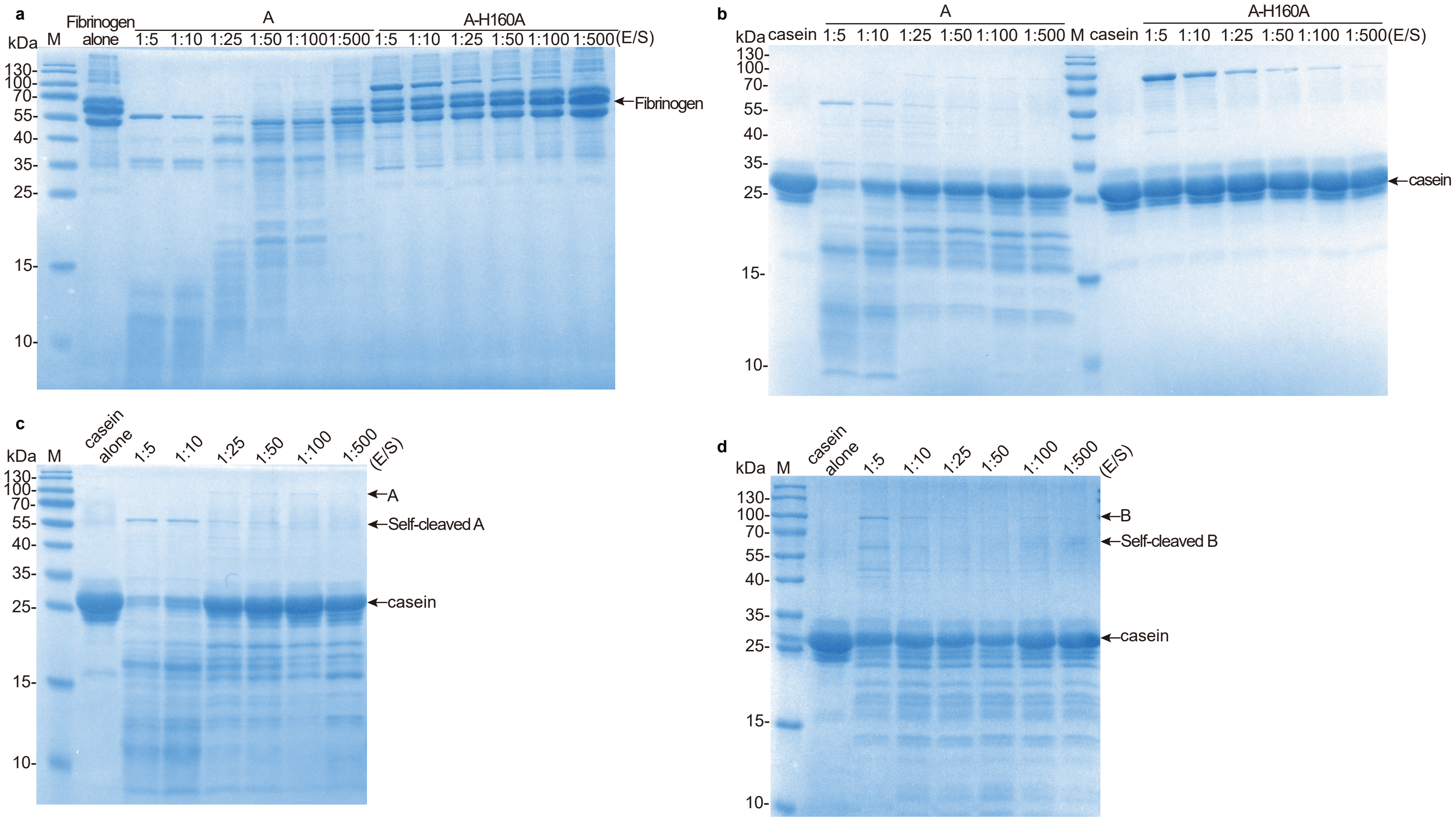

Supplementary Fig. 6

(a) Degradation of fibrinogen by peptidase A and A-H160A. Fibrinogen (final concentration 1.91 μg/μL) was mixed with different amounts of A or A-H160A in 115 μL buffer of 100 mM Tris-HCl, pH 7.4, 150 mM NaCl and 5 mM $CaCl_2$ and incubated for 30 min. The final concentration ratio of A or A-H160A (Enzyme, E) to fibrinogen (Substrate, S) were 1:5, 1:10, 1:25, 1:50, 1:100 and 1:500 (E/S, enzyme/substrate).

(b) Degradation of β-casein by peptidase A and A-H160A. β-casein (final concentration 0.87 μg/μL) was mixed with different amounts of A or A-H160A in 115 μL buffer of 100 mM Tris-HCl, pH 7.4, 150 mM NaCl and 5 mM $CaCl_2$ and incubated for 30 min. The final concentration ratio of A or A-H160A (Enzyme, E) to β-casein (Substrate, S) were 1:5, 1:10, 1:25, 1:50, 1:100 and 1:500 (E/S, enzyme/substrate).

(c) and (d) Degradation of β-casein by A (c) and B (d). β-casein (final concentration 0.87 μg/μL) was mixed with different amounts of A (c) or B (d) in 115 μL buffer of 100 mM Tris-HCl, pH 7.4, 150 mM NaCl and 5 mM $CaCl_2$ and incubated for 30 min. The final concentration ratio of A or B (Enzyme, E) to β-casein (Substrate, S) were 1:5, 1:10, 1:25, 1:50, 1:100 and 1:500 (E/S, enzyme/substrate). 10 μL samples were taken and mixed with 5×SDS loading buffer and denatured at 100 °C for 10 min to stop the reaction. The samples were then resolved by SDS-PAGE.

Supplementary Fig. 7

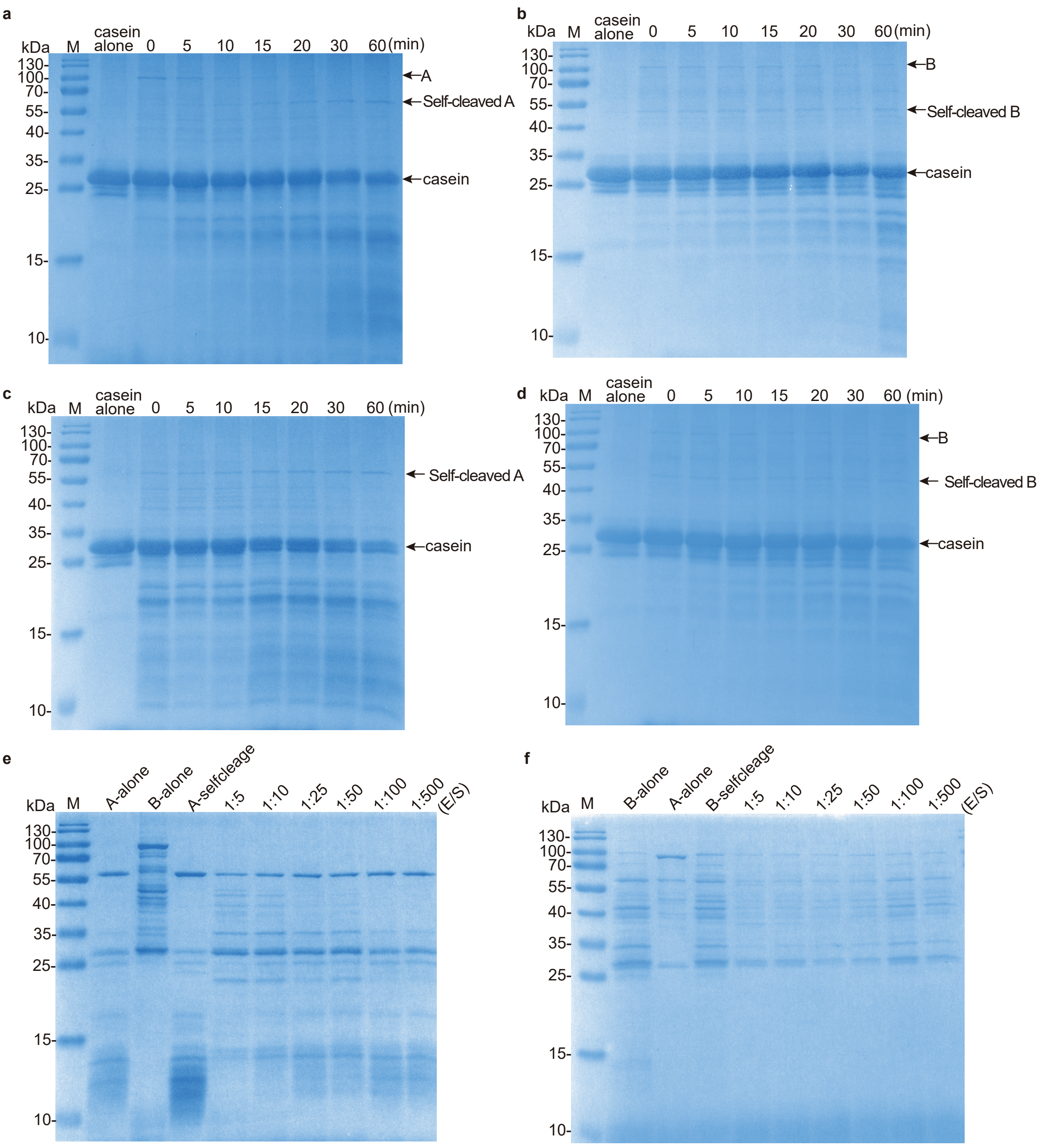

Supplementary Fig. 7

(a) and (b) Protease assays without self-cleavage of A (a) and B (b) (time course experiment). β-casein (final concentration 0.87 μg/μL) was preincubated with final concentration of 0.087 μg/μLA (a) and B (b) in115 μL buffer of 100 mM Tris-HCl, pH 7.4, 150 mM NaCl and 5 mM $CaCl_2$.

(c) and (d) Protease assays with self-cleavage of A (c) and B (d) (time course experiment). Final concentration 0.087 μg/μL A (c) and B (d) were preincubated at 37 °C for 20 min, then final concentration 0.87 μg/μL casein and 115 μL buffer of 100 mM Tris-HCl, pH 7.4, 150 mM NaCl and 5 mM $CaCl_2$ were added.

(e) and (f) Degradation of peptidase B (final concentration 0.26 μg/μL) by peptidase A (e) and degradation of peptidase A (final concentration 0.26 μg/μL) by peptidase B (f). The following conditions were examined: A-alone represents protein A; B-alone represents protein B; A (B)-self cleavage indicates that protein A (B) was incubated at 37 °C for 20 min. The remaining lanes represent various enzyme-to-substrate final concentration ratios (E/S) of 1:5, 1:10, 1:25, 1:50, 1:100, and 1:500.

Supplementary Fig. 8

N-terminal position 1

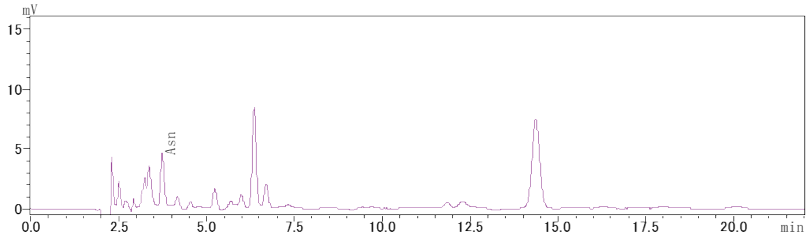

N-terminal position 2

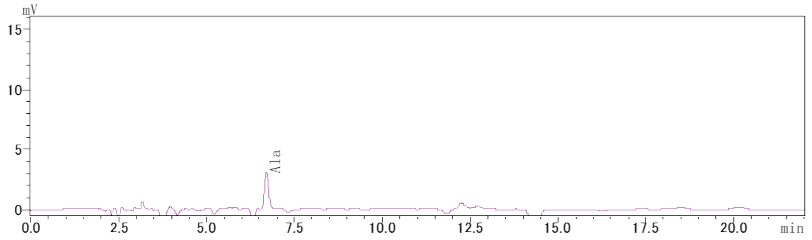

N-terminal position 3

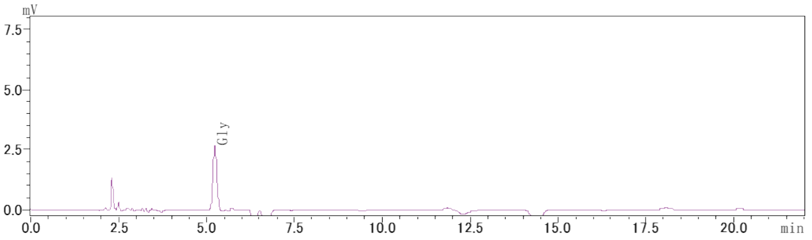

N-terminal position 4

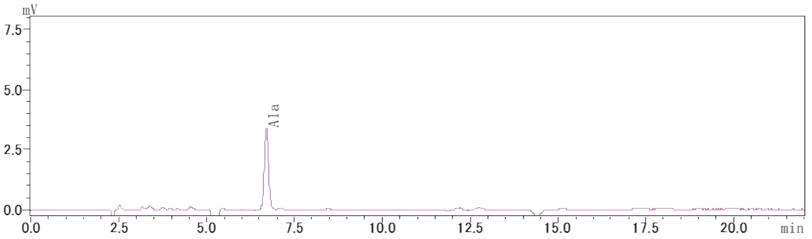

N-terminal position 5

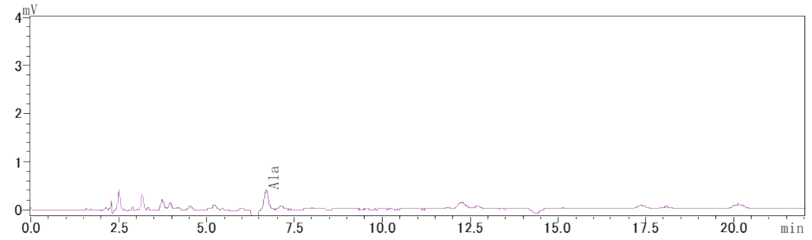

Supplementary Fig. 8

N-terminal sequencing results of protein A. Amino acid identification was performed using HPLC. In the HPLC chromatogram, a specific peak was produced for each amino acid. The sample peaks were compared with the retention times of standard amino acid peaks to identify the amino acid at the N-terminus.

Supplementary Fig. 9

N-terminal position 6

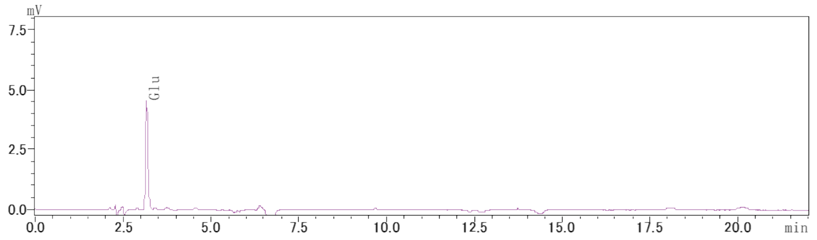

N-terminal position 7

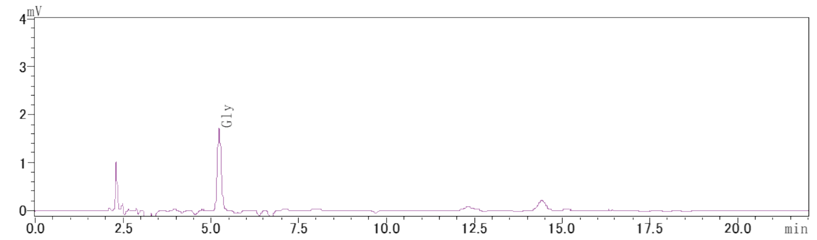

N-terminal position 8

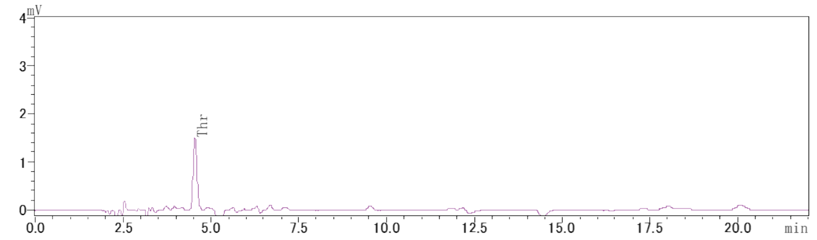

N-terminal position 9

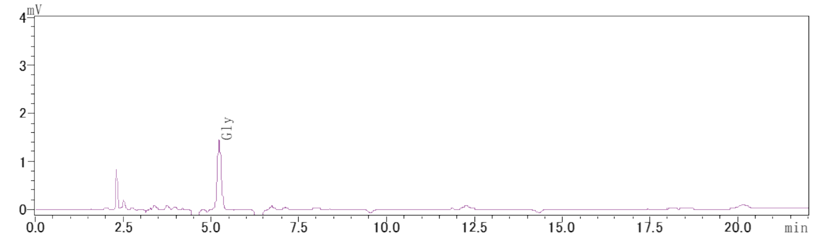

N-terminal position 10

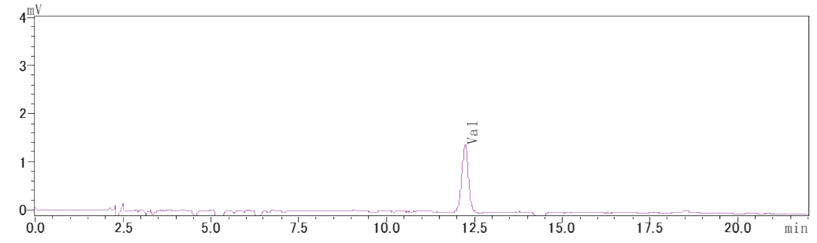

Supplementary Fig. 9

N-terminal sequencing results of protein A. Amino acid identification was performed using HPLC. In the HPLC chromatogram, a specific peak was produced for each amino acid. The sample peaks were compared with the retention times of standard amino acid peaks to identify the amino acid at the N-terminus.

Supplementary Fig. 10

a

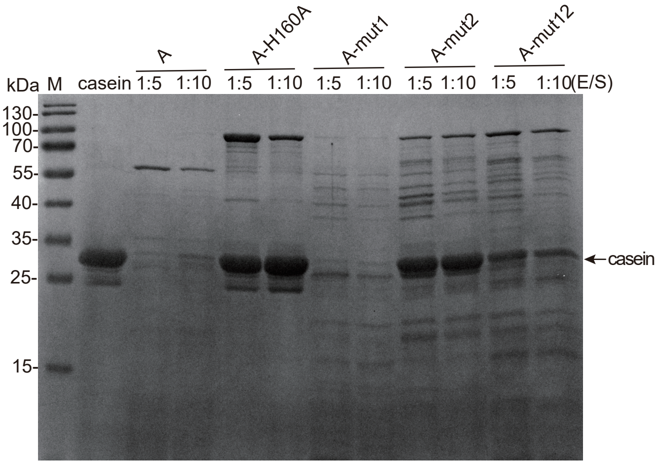

b

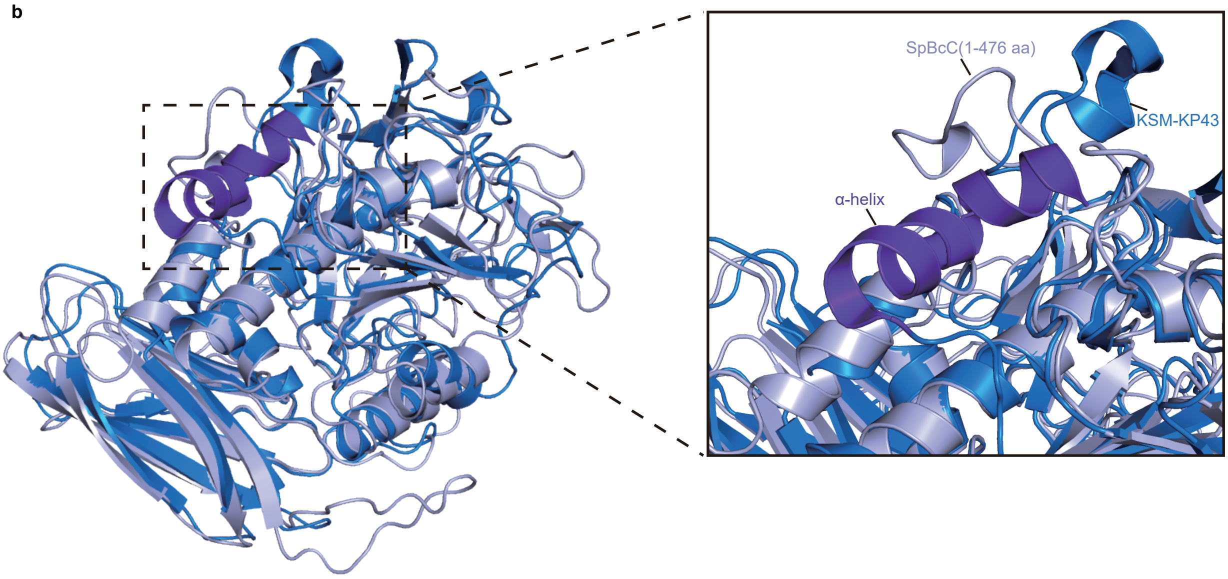

Supplementary Fig. 10

(a) The black and white image of degradation of β-casein by A, A-H160A, A-mut1, A-mut2, and A-mut12. Detailed information about the band is provided in Fig. 8d.

(b) Structural comparison of SpBcC (1-476aa) and a subtilisin-like alkaline serine protease, KP-43, from *Bacillus sp.* KSM-KP43 (PDB code: 1WMD). The structure of SpBcC (1-476aa) S8-peptidase domain was superposed with the KP-43 (PDB code: 1WMD). The structure of SpBcC (1-476aa) S8-peptidase domain was colored in light blue. The structure of KP-43 S8-peptidase domain was colored in blue. The α-helix was colored in purple. Structural figures were made using PyMOL (Schroedinger).

Supplementary Table 1 Identification results of peptidases

Accession number	Gene number	Protein name	Bacteria
QHN65200.1	DBX24_04465	SpBcA	*Bergeyella cardium* HPQL
QHN65156.1	DBX24_04215	SpBcB	*Bergeyella cardium* HPQL
QHN66091.1	DBX24_02695	SpBcC	*Bergeyella cardium* HPQL
QHN65150.1	DBX24_04180		*Bergeyella cardium* HPQL
QHN65828.1	DBX24_08020		*Bergeyella cardium* HPQL
QHN64674.1	DBX24_01580		*Bergeyella cardium* HPQL

(Note: QHN65200.1, QHN65156.1 and QHN66091.1 refer to SpBcA, B and C; The sequences of QHN65150.1 and QHN65200.1 are identical; QHN65828.1 and QHN64674.1 only contain the S8 peptidase domain.)

Supplementary Table 2 Strains and plasmids used in this study

**Strains, plasmids, and primers**	**Descriptions**	**Source**
Strains		
B. cardium HPQL	*Bergeyella cardium* wide-type strain	Preserved in the laboratory
Escherichia coli DH5α	Competent cell	Solarbio
Escherichia coli BL21(DE3)	Competent cell	Solarbio
Plasmids		
pGEX-6p-2	Expression vector	Addgene
pGEX-6p-2- SpBcA	pGEX-6p-2 containing SpBcA	This study
pGEX-6p-2- SpBcB	pGEX-6p-2 containing SpBcB	This study
pGEX-6p-2-SpBcC	pGEX-6p-2 containing SpBcC	This study
pGEX-6p-2-A	pGEX-6p-2 containing A	This study
pGEX-6p-2-B	pGEX-6p-2 containing B	This study
pGEX-6p-2-A-H160A	pGEX-6p-2 containing A-H160A	This study
pGEX-6p-2-B-H160A	pGEX-6p-2 containing B-H160A	This study
pGEX-6p-2-SpBcA (104-601aa)	pGEX-6p-2 containing SpBcA (104-601aa)	This study
pGEX-6p-2-A-mut1	pGEX-6p-2 containing A-mut1	This study
pGEX-6p-2-A-mut2	pGEX-6p-2 containing A-mut2	This study
pGEX-6p-2-A-mut12	pGEX-6p-2 containing A-mut12	This study

Supplementary Table 3 Primers used in this study

**Primers**	**Characteristics**
SpBcA-F	5’-ATATGGATCCATGAGAAAGTCTACATTAGAG-3’ (BamHI site underlined)
SpBcA-R	5’-ATATCTCGAGTTACTGAACGATGAGTTTAGC-3’ (XhoI site underlined)
SpBcB-F	5’-ATATGGATCCATGACTAAATCTATATTT-3’ (BamHI site underlined)
SpBcB-R	5’-ATATCTCGAGTTACTGAACGATGAGCTTAGC-3’ (XhoI site underlined)
SpBcC-F	5’-ATATGGATCCATGCGTGTCTTTGAATGGGAC-3’ (BamHI site underlined)
SpBcC-R	5’-ATATCTCGAGCTACTTGATGATGAGCTTAGC-3’ (XhoI site underlined)
A-F	5’-ATATGGATCCATGCAGAATGCCCAAGAGG-3’ (BamHI site underlined)
A-R	5’-ATATCTCGAGTTAACCATTGTTAATTCCCGTTGC-3’ (XhoI site underlined)
B-F	5’-ATATGGATCCATGCAGAATGCAGAGGAAATAA-3’ (BamHI site underlined)
B-R	5’-ATATCTCGAGTTAACCTGTATTAATTCCCGTTGC-3’ (XhoI site underlined)
SpBcA (104-601aa)-F	5’-ATATGGATCCATGGGAGCAGCAGAAGGTAC-3’ (BamHI site underlined)
SpBcA (104-601aa)-R	5’-ATATCTCGAGTTAACCATTGTTAATTCCCGTTGCA-3’ (XhoI site underlined)
A-H160A-F	5’-CACGGCTTAATAGCGCCTCTACCCATGTGGCG-3’
A -H160A-R	5’-CGCCACATGGGTAGAGGCGCTATTAAGCCGTG-3’
B-H160A-F	5’-AATTCCCTAATAGCgcCTCTACCCATGTGGCG-3’
B -H160A-R	5’-CGCCACATGGGTAGAGgcGCTATTAGGGAATT-3’
A-mut1-F	5’-GCTTCCCTTTGTACTATGTAgCGgcCgcTGCTGGAGCAGCAGAAGGTACGGG-3’
A-mut1-R	5’-CCCGTACCTTCTGCTGCTCCAGCAgcGgcCGcTACATAGTACAAAGGGAAGC-3’
A-mut2-F	5’-CGGGAGTTTCAAGGCTTCAGgCGgcCgCAGcAgcGGAACTTCAAGGAGAAGGTAT-3’
A-mut2-R	5’-ATACCTTCTCCTTGAAGTTCCgcTgCTGcGgcCGcCTGAAGCCTTGAAACTCCCG-3’
