# Supplementary material for: Functional study of Bergeyella cardium KP-43 subfamily peptidases as putative T9SS cargo
Source: Commun Biol. 2025 Apr 9;8:586. doi: 10.1038/s42003-025-07996-y (PMC11982257; doi:10.1038/s42003-025-07996-y)
Supplement: Supplementary file 2 — Description of Additional Supplementary Files [file 42003_2025_7996_MOESM2_ESM.docx]

Description of Additional Supplementary Files

**File Name:** Supplementary Data 1

**Descriptions:** The statistical source data for graphs in Fig. 3, Fig.4e, f, Fig. 5c-f, Fig. 8e, f and Fig. 10d in the paper.

**File Name:** Supplementary Data 2

**Descriptions:** The unprocessed gel images as source data for the gel images in the paper.

**File Name:** Supplementary Data 3-7

**Descriptions:** The original N-terminal sequencing data corresponding to Supplementary Fig. 8 in the paper.

**File Name:** Supplementary Data 8-12

**Descriptions:** The original N-terminal sequencing data corresponding to Supplementary Fig. 9.
