# Supplementary material for: Functional study of Bergeyella cardium KP-43 subfamily peptidases as putative T9SS cargo
Source: Commun Biol. 2025 Apr 9;8:586. doi: 10.1038/s42003-025-07996-y (PMC11982257; doi:10.1038/s42003-025-07996-y)
Supplement: Supplementary file 4 — Supplementary Data 2 [file 42003_2025_7996_MOESM4_ESM.pdf]

Figure 2

d

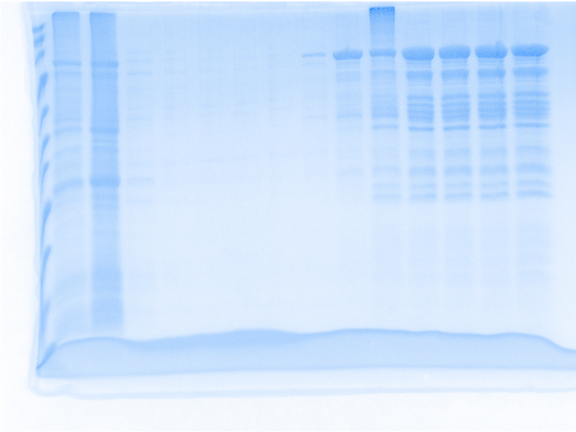

f

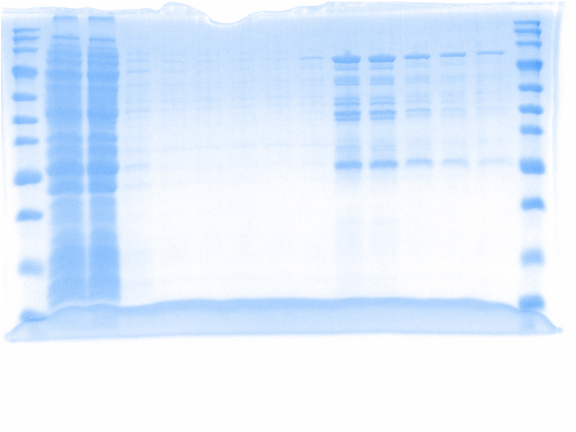

Figure 4

c

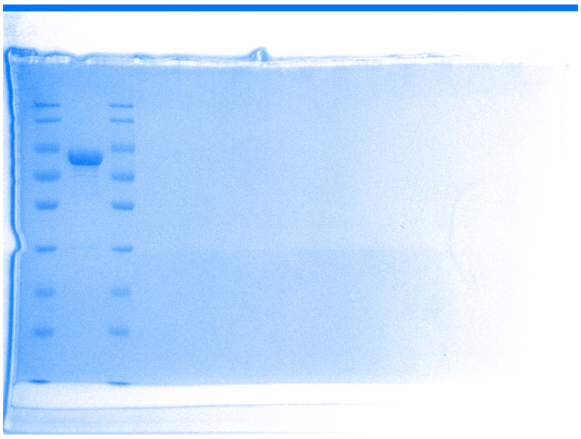

d

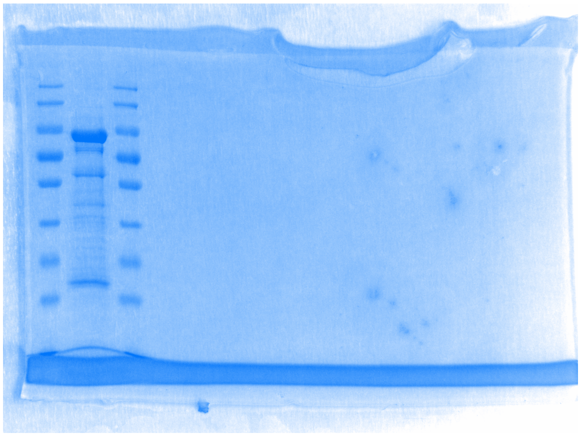

Each image is scaled down by 80%, with no modifications made other than the resizing.

Figure 5

a

b

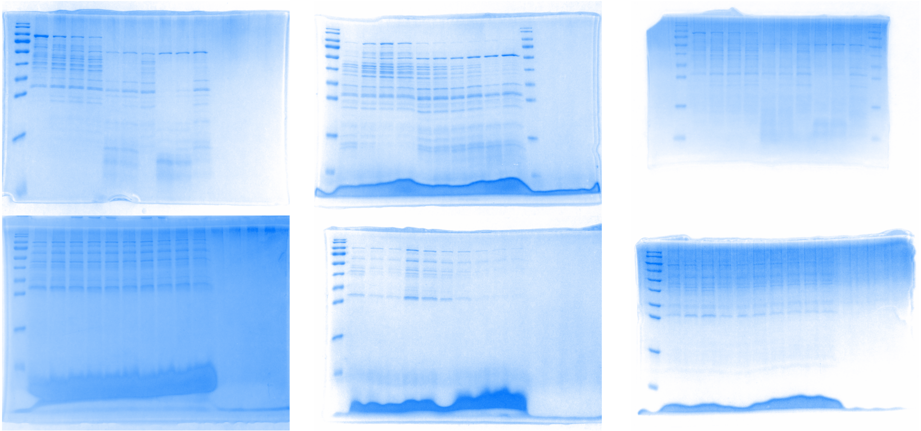

Figure 6

a

b

c

d

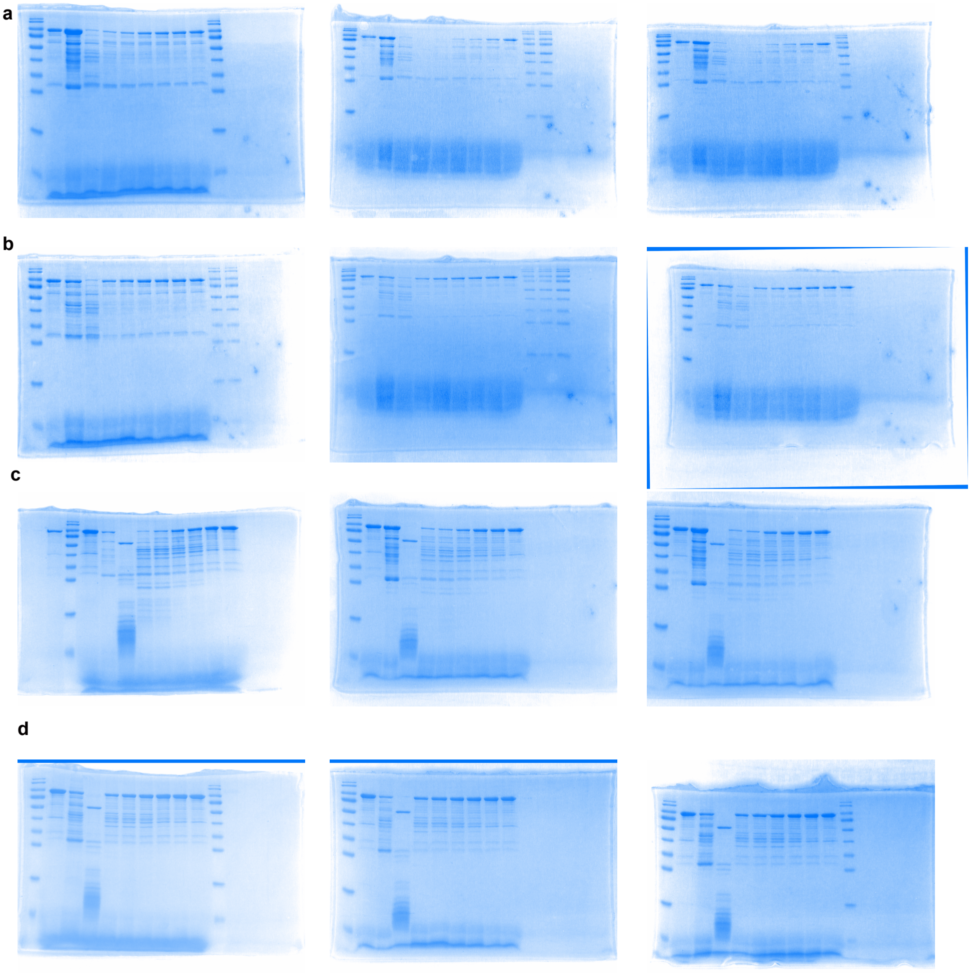

Each image is scaled down by 40%, with no modifications made other than the resizing.

Figure 8

a

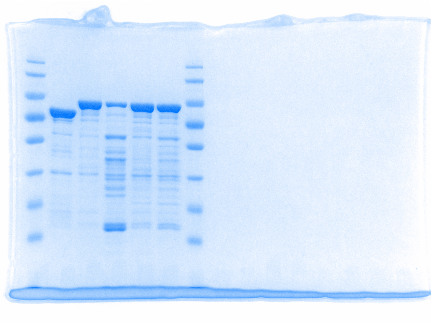

b

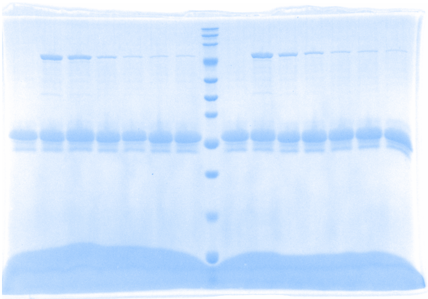

d

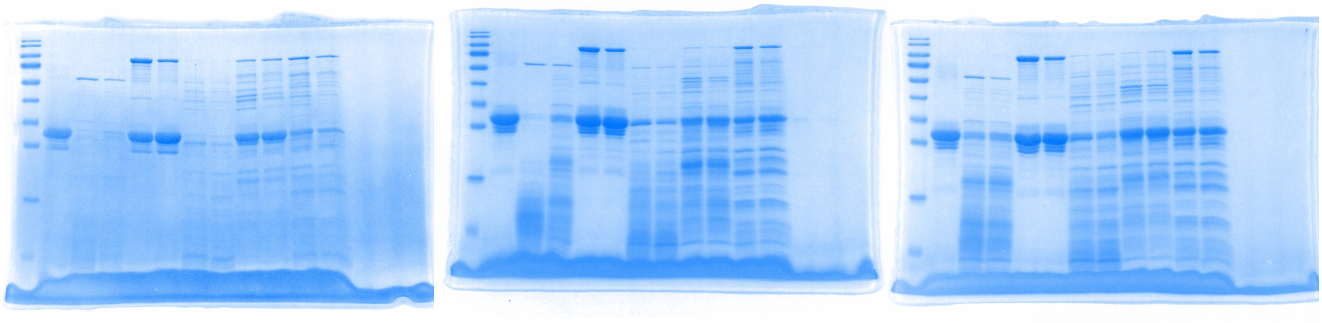

Each image is scaled down by 60%, with no modifications made other than the resizing.

Figure 9

a

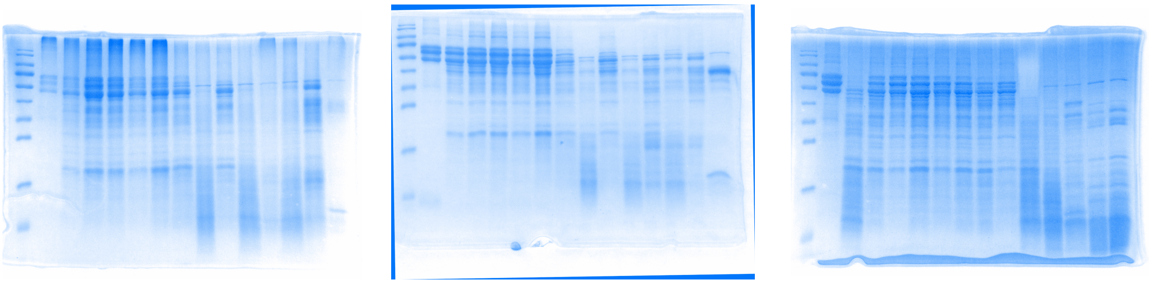

b

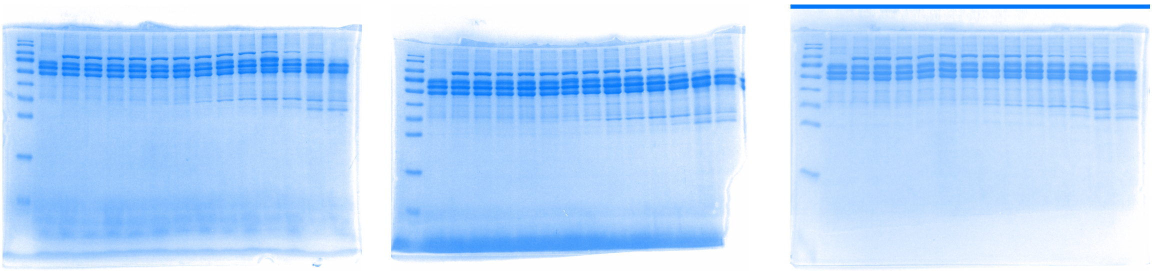

c

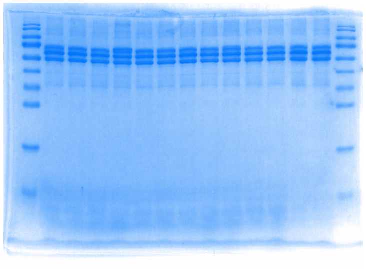

d

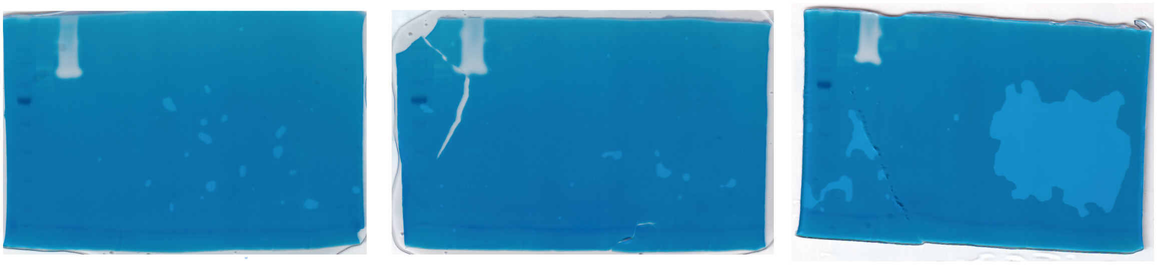

e

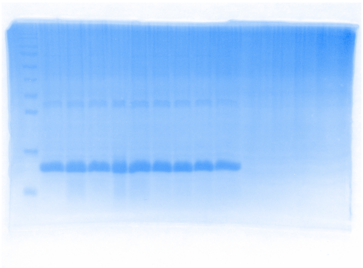

Each image is scaled down by 50%, with no modifications made other than the resizing.

Figure 10

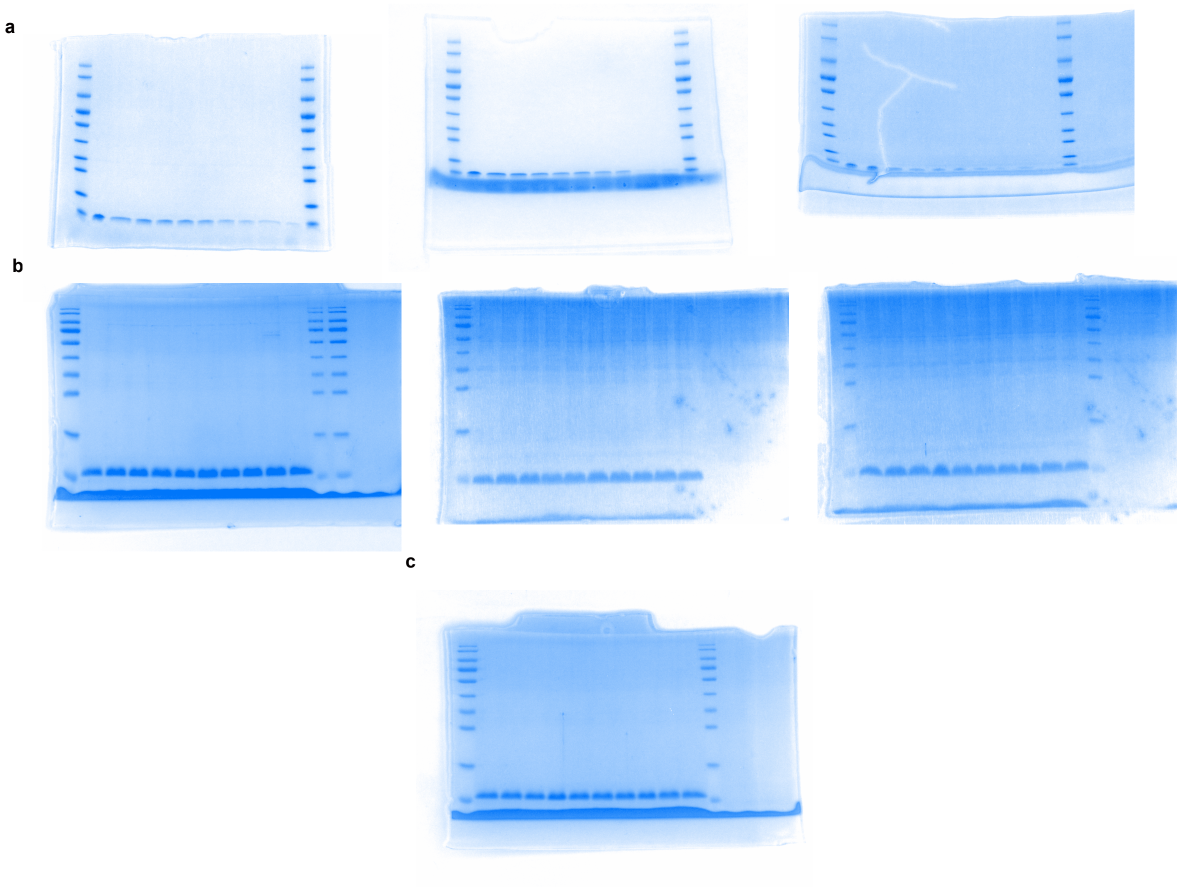

Each image is scaled down by 50%, with no modifications made other than the resizing.

Supplementary Figure 2

a

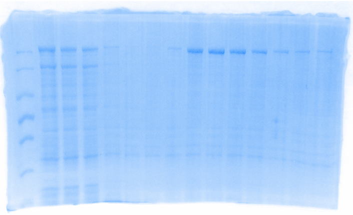

b

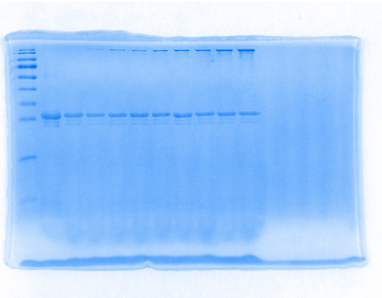

Supplementary Figure 3

a

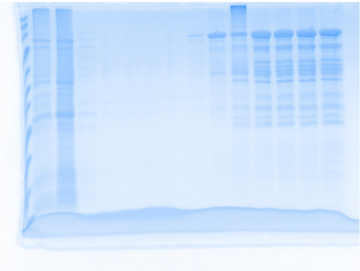

c

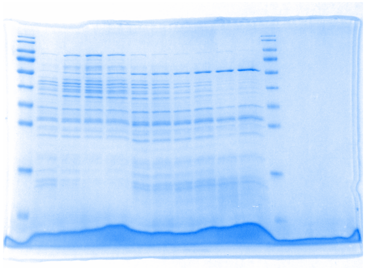

Supplementary Figure 4

a

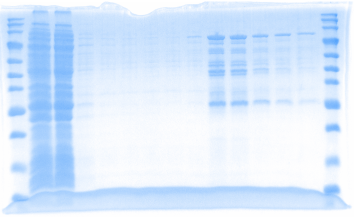

Supplementary Figure 6

a

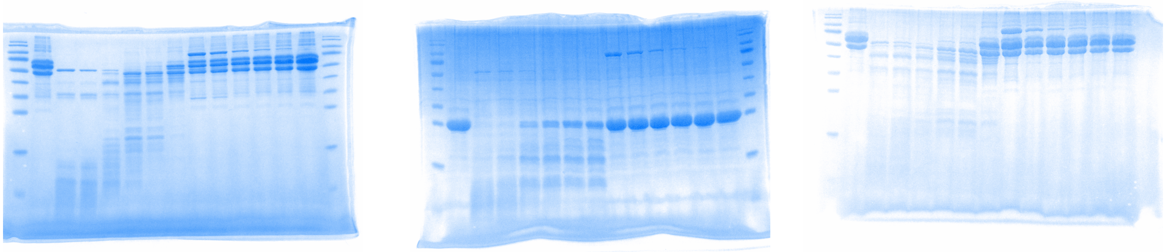

b

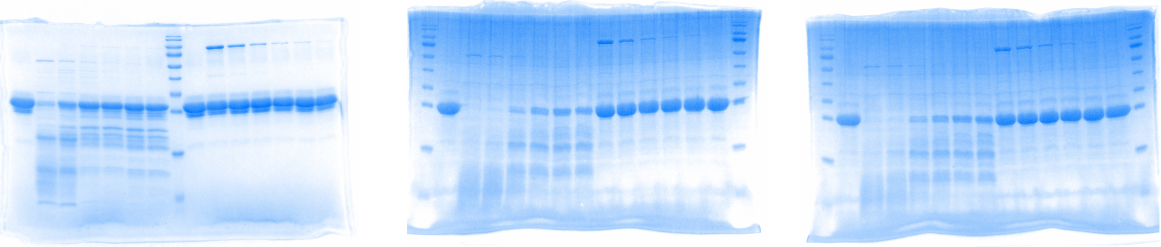

c

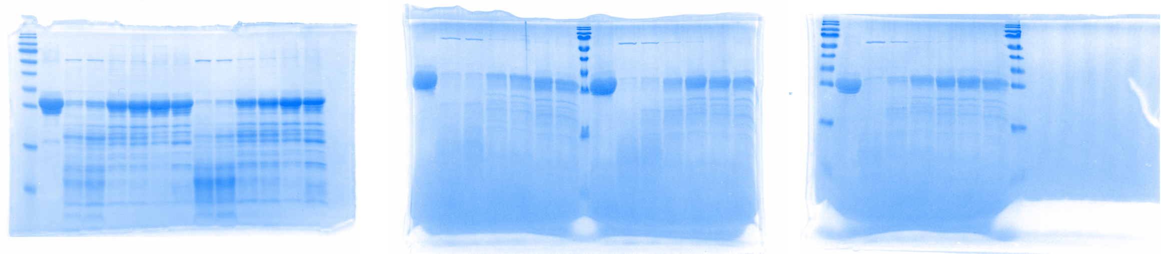

d

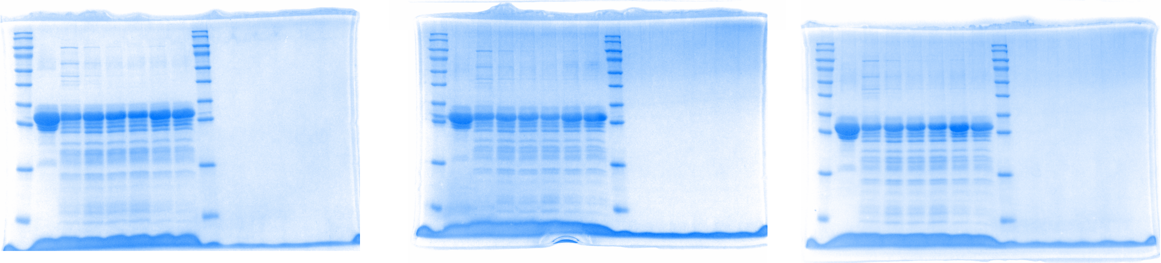

Each image is scaled down by 50%, with no modifications made other than the resizing.

Supplementary Figure 7

a

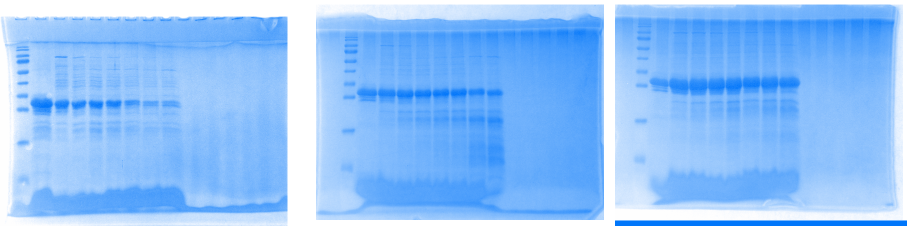

b

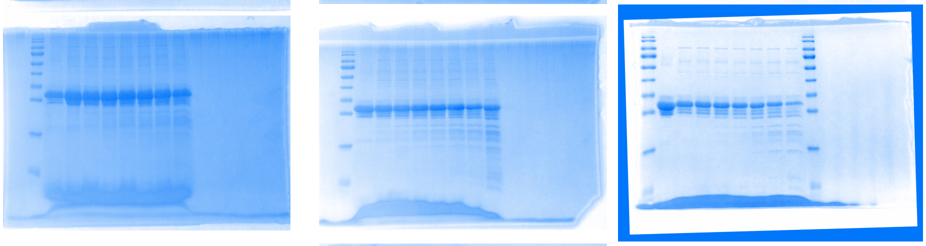

c

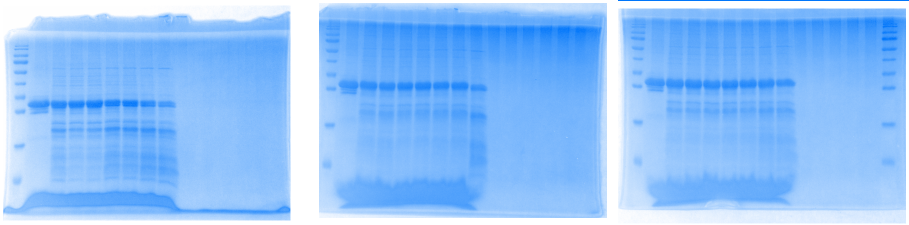

d

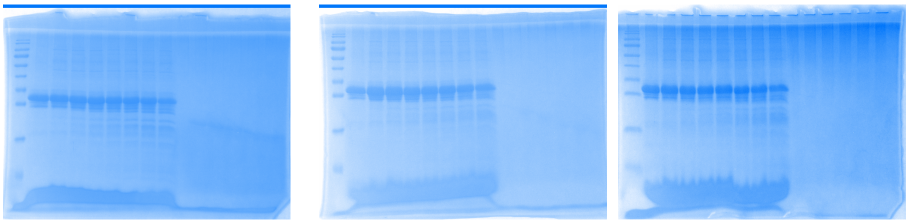

e

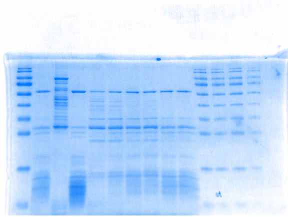

f

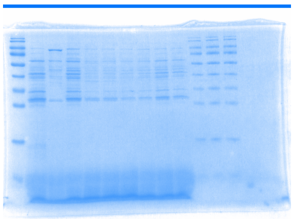

Supplementary Figure 10

a

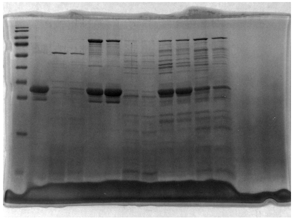

Each image is scaled down by 40%, with no modifications made other than the resizing.
